# ﻿Checklist of the diatoms (Bacillariophyta) from Lake Naivasha, Kenya, with some historical notes

**DOI:** 10.3897/phytokeys.224.98168

**Published:** 2023-04-07

**Authors:** Christine Cocquyt, Dirk Verschuren

**Affiliations:** 1 Research Department, Meise Botanic Garden, 1860 Meise, Belgium Ghent University Ghent Belgium; 2 Limnology Unit, Ghent University, 9000 Gent, Belgium Meise Botanic Garden Meise Belgium

**Keywords:** biodiversity, Crescent Island Crater, East Africa, Lake Oloidien, Lake Sonachi

## Abstract

Lake Naivasha is one of only two large freshwater lakes in the Eastern Rift Valley of Kenya, East Africa. Together with its satellite lakes Crescent Island Crater, Oloidien and Sonachi, it comprises a great variety of pelagic and benthic habitats for aquatic biota, and its sediment record represents a unique archive of past climate change and long-term ecosystem dynamics in equatorial East Africa. This is particularly so because local paleoenvironmental reconstructions can be checked against historical data on the composition of aquatic fauna and flora collected in Lake Naivasha since the early 20^th^ century. Some of the most prominent biological proxies for reconstructing past changes in lakes are diatoms (Bacillariophyta), a group of unicellular autotrophic eukaryotes of which the siliceous skeletons (valves) preserve well in lake sediments and are good indicators for, among others, climate-driven changes in salinity. However, diatom taxonomy and species concepts have changed a lot in recent decades, making it sometimes difficult for non-taxonomists to know which species are concerned in different published studies. This paper provides the currently accepted taxonomic names of the 310 specific and infraspecific diatom taxa reported from Lake Naivasha and its satellite lakes to date, together with their synonyms used in literature concerning these lakes as well as other, commonly used synonyms. Further, a short overview is given of the history of diatom research conducted on materials from Lake Naivasha and its satellite lakes. The present checklist may facilitate the identification and interpretation aspects of future diatom studies on the wider Lake Naivasha ecosystem and on other East African lakes that are less well studied.

## ﻿Introduction

**Figure 1.**
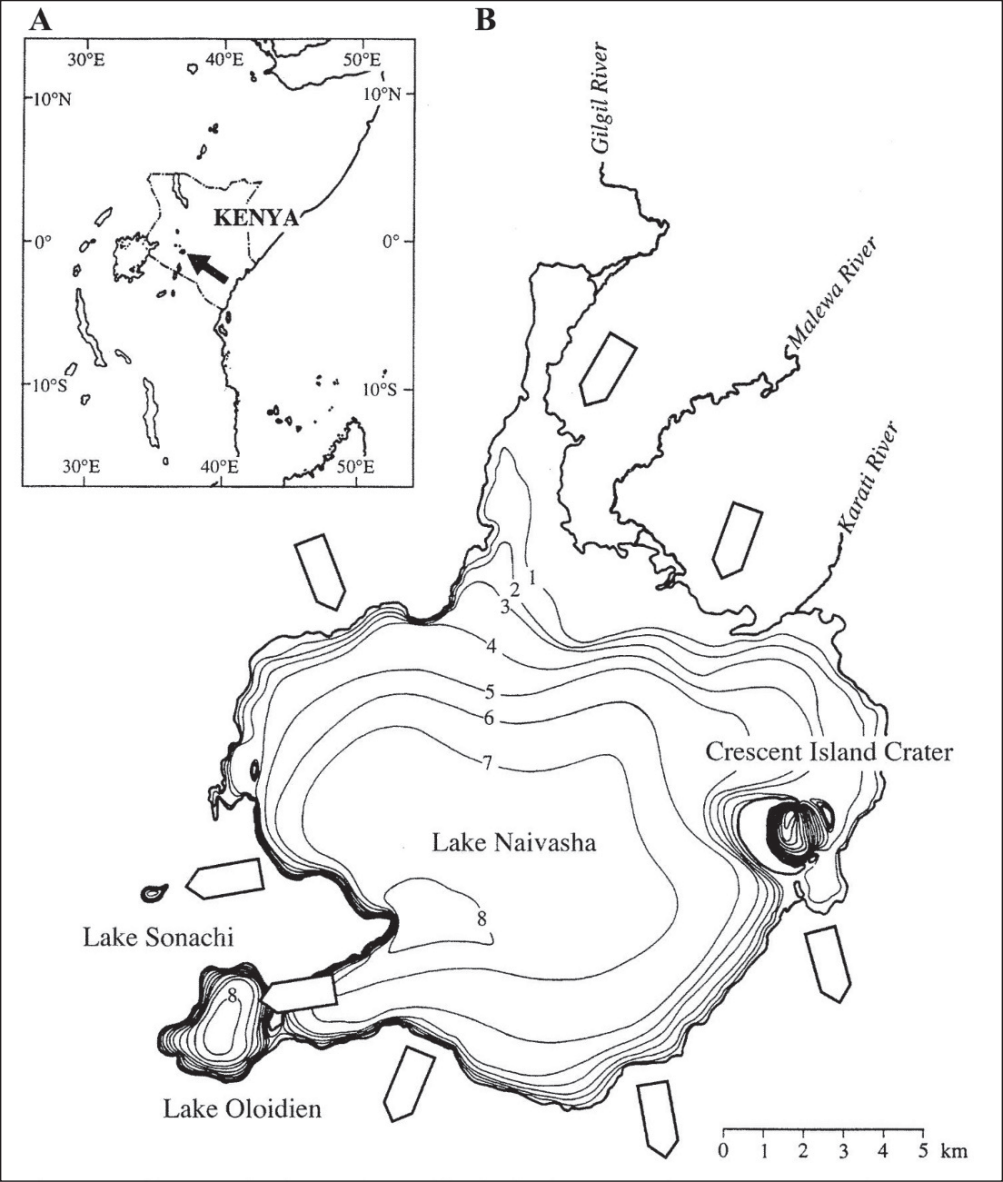
**A** location of Lake Naivasha in Kenya, East Africa **B** bathymetric map of Lake Naivasha and its satellite Lakes Oloidien, Sonachi and Crescent Island crater, relative to a lake-surface elevation of 1885.8 m above sea level. White arrows show the direction of the groundwater flow. From Verschuren et al. (2000b), as modified after Gaudet and Melack (1981) and Åse et al. (1986).

Lake Naivasha is located at about 1885 m a.s.l. (above sea level) in the central valley of the Eastern (Gregory) Rift in Kenya between 0°43'08"S and 0°49'57"S and between 36°16'54"E and 36°25'46"E (Fig. 1). With a surface area fluctuating around 135 km^2^, Lake Naivasha is, besides Lake Baringo, the only large freshwater lake in Kenya’s portion of the Eastern Rift Valley, and consequently an important source of freshwater in the rift-valley region. It has two smaller satellite lakes, which, depending on the lake level, can be confluent with it or separated by a narrow sill: Lake Oloidien with a surface area of 5.1 km^2^ at its southwestern corner, and Crescent Island Crater (1.9 km^2^) along its eastern shore. Its third satellite lake is Lake Sonachi, also referred to as Crater Lake (e.g., Rich 1932) or Green Crater Lake (e.g., Damnati et al. 1991), is a very small saline crater lake (0.14 km^2^) situated to the west and receiving underground water supply from the main basin of Lake Naivasha (MacIntyre and Melack 1982; Verschuren 1999a).

While the main basin of Lake Naivasha is hydrologically open, fed by the Malewa and Karati Rivers in the east and the Gilgil River in the north, and groundwater outflow to the south and the southeast, Lake Oloidien is hydrologically closed (Gaudet and Melack 1981; Verschuren et al. 2000b). Without its own river inflow, this lake depends on local rainfall and either direct confluence or subsurface inflow from Lake Naivasha (Verschuren et al. 1999a, 2000b). Crescent Island Crater Lake is hydrologically open through its direct confluence with the main lake. Only during periods of severe lowstands, when the connection with the main lake is fully interrupted, does it become a hydrologically closed system (Verschuren 2001; Van der Meeren et al. 2019).

During lake highstands, such as first recorded in 1897 but also the present-day situation after two episodes of strong transgression in 2011, 2012 and 2020, Lake Oloidien is broadly confluent with Naivasha and contains fresh water. However, when separated from Lake Naivasha during lake lowstands, it develops higher salinity because it then depends on local rainfall and subsurface inflow, while water losses are almost entirely due to evaporation (Verschuren et al. 2000b). Lake Sonachi is normally a strongly saline-alkaline (‘soda’) lake, but substantial changes in salinity during past episodes of wetter and drier climate conditions have also been reported in historical times (Verschuren et al. 1999a). This implies that freshwater as as well as inland saline communities of aquatic biota, among others of diatoms, can be found in the lakes of this aquatic system, making Lake Naivasha and its satellite lakes ideal for paleolimnological research involving both climate reconstruction and long-term ecological dynamics (Verschuren et al. 1999a, b, 2000a, 2000b; Mergeay et al. 2011; Van der Meeren et al. 2019; Van der Meeren and Verschuren 2021).

However, diatom taxonomy and species concepts have changed a lot since species description in this group of unicellular algae started in the 19^th^ century. After a period of species lumping in the 20^th^ century, the end of the last century saw the erection of new and restoration of many formerly described genera and species mainly due to better microscopes. Moreover, many new species were discovered, including tropical African taxa which had previously been assigned to European and/or North American taxa due to the use of identification guides from these north-temperate regions. This makes it often very difficult for non-taxonomists to know exactly which diatom species are involved in the older and the more recent literature on Lake Naivasha. Because of the importance of Lake Naivasha in East African paleoecological and paleoclimate studies, and the fact that diatoms have proven to be good indicators (so-called ‘proxies’) for changes in salinity (and nutrients) and reconstruction of past environmental situations, we found it opportune to make a checklist of all the diatoms reported up to now from Lake Naivasha and its three satellite lakes. Notably, the present checklist covers both recent phytoplankton and periphytic collections as well as fossil diatom valves recovered from sediment cores. The current taxonomically accepted names are provided together with their synonyms used in different studies as well as references to the publications or materials in case of unpublished results.

## ﻿Material and methods

The material used for the present checklist is twofold. On the one hand, all literature data known to us, published between 1932 and the present, among others Rich (1932), Bachmann (1938), Richardson and Richardson (1972), Gasse (1986), Verschuren et al. (1999a), Verschuren et al. (1999b), Verschuren et al. (2000a), Verschuren et al. (2000b), Cocquyt and De Wever (2002), Stoof-Leichsenring et al. (2011), Stoof-Leichsenring et al. (2012) and Owino et al. (2020). On the other hand, counts of fossil diatom assemblages carried out on sediment cores from Naivasha, Crescent Island Crater, Oloidien and Sonachi, whose results are either unpublished, or were used for publications focusing on paleoenvironmental reconstruction that did not include full species lists.

The sediment sequences covered in this paper are listed in Table 1, with citation of the publication providing the most detailed information on their collection and characteristics.

**Table 1.** Sediment cores from Lakes Naivasha, Crescent Island, Oloidien and Sonachi included in the checked list, with the date of coring, the core length and age, and the reference to the publication providing the most detailed information.

Core	Lake	Coring date	Core length	Core age	Reference
NC93	Crescent Island Crater	08/1993	8.22 m	ca. 200 AD to Present	Verschuren 2001
(NC93.1-S + NC93.1-L)					
NS93.2-F	Sonachi	08/1993	0.37 m	ca. 1750 AD to Present	Verschuren 1999a
NM93.1-S	main basin of Naivasha	08/1993		ca. 1800 AD to Present	Verschuren 1999b
NM91.1-S	main basin of Naivasha	08/1991		ca. 1800 AD to Present	Verschuren 1996
NO91.1-S:	Oloidien	08/1991	0.92 m	ca. 1800 AD to Present	Verschuren 1999b
NC20	Crescent Island Crater	01/2022	23.0 m	ca. 7000 BP to Present	Nguyen 2022
(NC20-3G + NC20-1P)					

All sediment cores were recovered from an anchored boat or platform using a combination of gravity coring and piston coring, except for NS93.2-F which was recovered using freeze coring (Verschuren 1999a).

## ﻿Historical overview of diatom sampling and studies in Lake Naivasha

The first reports on diatoms from Lake Naivasha and its satellite lakes date back to the 1930s. Rich (1932) investigated samples collected between 18 April and 7 July 1929 by Miss Penelope Jenkin during the Percy Sladen Expedition to Kenya’s Rift Valley Lakes (Jenkin 1936). From the 13 Naivasha samples she investigated, she only reported diatoms in three samples from near the mouth of the Gilgil River at the north end of the lake. In sample number 135 (surface) and number 193 (water over *Ceratophyllum*) Rich (1932) mentioned one and three diatom taxa respectively, and 30 diatom taxa in sample number 138 (mud) of which a diatom preparation was made. Besides the 30 taxa from the main basin of Naivasha, Rich (1932) reported one diatom, *Rhopalodiaventricosa* from one of the two samples collected in Lake Sonachi (referred to as ‘Crater Lake’). Rich (1933) expanded the species list of Lake Naivasha by two taxa observed in samples taken in November 1930 and February 1931.

During the “Mission scientifique de l’Omo”, organized by R. Jeannel and C. Arambourg, plankton samples from some Rift Lakes in Kenya were taken by hydrobiologist P.A. Chappuis, at the end of this expedition on the return from the Omo Valley (Ethiopia) to Mombasa (Kenya) from where the expedition members embarked back to France (Lester 1933). From the phytoplankton sample taken in Lake Naivasha on 12 April 1933 and which contained a lot of detritus from plant remains, Bachmann (1938) reported 14 diatom taxa. From the sample taken in ‘Crater Lake’ (= Lake Sonachi), which was dominated by *Arthrospiraplatensis* Gomont [as *Spirulinaplatensis* (Gomont) Geitler], Bachmann (1938) mentioned 4 diatom species.

By the end of the 1930s, 43 diatom species and infraspecific taxa were known from Lake Naivasha, distributed among 15 genera sensu lato; for Lake Sonachi this was only 5 species, belonging to five genera. However, *Rhopalodiaventricosa* was the only species from Lake Naivasha reported by both Rich (1932) and Bachmann (1938). About thirty years later, Richardson and Richardson (1972) reported 112 diatom taxa from a 28-meter long sediment sequence from Crescent Island Crater, covering the last ca. 9000 years and obtained by combining mutiple sediment cores taken between 30 December 1960 and 2 January 1961, and analyzed at 20-cm intervals. These taxa, 96 species and 16 varieties, are distributed among 25 genera, based on the taxonomy used. Of the 96 species, 15 are referred to as “cf.” and 5 as “sp.”.

Gasse (1986) studied six phytoplankton net samples from Lake Naivasha collected by herself on 5 December 1979 and by J. Kalff on 8 February, 18 March, 19 April, 2 May and June 1980, as well as one littoral mud sample collected by herself on 5 December 1979 and two bottom samples collected by C. Barton. From Crescent Island Crater she analyzed one phytoplankton net sample and one bottom mud sample collected by herself and C. Barton respectively. Finally, Gasse (1986) reported on three samples from Lake Sonachi collected on 6 December 1979: a phytoplankton net sample, littoral mud and scrapings from dead trees. From this total of 11 samples, Gasse (1986) reported 20 genera, 70 species, more than 14 varieties, two forms, one taxon with confer (“cf.”), one taxon with affinity (“aff.”) and two unknown species (“sp.”). The exact number of varieties cannot be tracked down as it is not clear how many are included in the mentioned “and varieties”.

In the 1990s, the growing interest in climate change in East Africa and worldwide led to several coring campaigns in Lake Naivasha and its satellite lakes followed by intensive paleolimnological studies of the recovered sediment cores (Verschuren et al. 1999a, 1999b, 2000a, 2000b). Diatoms were one of the paleoecological proxies studied in a 8.22-m long composite sediment core (NC93) from Crescent Island Crater covering the last ca. 1650 years (Verschuren et al. 2000a; Van der Meeren et al. 2019), and in a shorter sediment core of 71-cm from Oloidien (NO91.1-S) covering the last ca. 200 years (Verschuren et al. 1999b, 2000b). For Lake Sonachi diatom studies were performed on 50 samples from a 37.2-cm freeze-core (NS93.2-F) collected in 1993 (Verschuren et al. 1999a). Diatoms from a sediment core taken in the main basin of Naivasha (NM93.1-S) were also investigated by one of us (CC) but these results have not been published to date.

The above mentioned paleoecological studies inspired Cocquyt and De Wever (2002) to study the epiphytic diatom communities in Lake Naivasha and its satellite lakes. For this purpose, herbarium specimens of aquatic plants collected in Lake Naivasha between 1909 and 1933, and kept in the collections of the Meise Botanic Garden (BR), were investigated: *Nymphaeacaerulea* Savigny, *Potamogetonpectinatus* Linnaeus, *P.schweinfurthii* A.Bennett and *Najashorrida* A.Braun ex Magnus. Additional materials of *Nymphaeacaerulea* and *Cyperuslaevigatus* Linnaeus collected in 1999 in Lake Naivasha and Lake Sonachi respectively, were studied (Cocquyt and De Wever 2002).

Based on microscopic (i.e., morphological) analyses of fossil diatoms in core NSA-3 from the main basin of Lake Naivasha, Stoof-Leichsenring et al. (2011) reported 39 diatom species, while in follow-up molecular analyses these authors could identify 28 different diatom haplotypes in bulk sediment samples (Stoof-Leichsenring et al. 2012). All haplotypes that differed < 8% to a species-specific GenBank sequence (corresponding to a similarity of 92–100%) were assigned to that species. Haplotypes with a similarity below 92% to any reference sequence, were not assigned to a species, but to the respective diatom family. This implied that the genetic survey did not reveal all species morphologically identified. However, all genetic information and morphological data were highly correlated but not fully identical (Stoof-Leichsenring et al. 2012). It is clear that the African diatom flora is still not well known either morphologically or molecularly.

This brief overview of the diatom research on Lake Naivasha and its satellite lakes covers only taxonomic relevant publications for diatoms (Table 2). The numerous and important studies done on phytoplankton biomass, dynamics, chlorophyll, etc. and papers on algae other than diatoms, such as Kalff and Watson (1986), Harper et al. (1993, 2003) and Ballot et al. (2009), are not included in the present overview. However, the diatom species names mentioned in those ecological studies concern the most common diatoms, of which the taxon names can be found in this checklist either as a currently accepted name or as a synonym.

**Table 2.** Overview of the number of diatom species and infraspecific taxa reported in the most important publications from Lake Naivasha and its satellite basins mentioning morphological diatom identifications.

Lake	Naivasha	Crescent Island Crater	Oloidien	Sonachi
Rich (1932)	29	-	-	1
Bachmann (1938)	14	-	-	4
Richardson and Richardson (1972)	-	102	-	-
Gasse (1986)	58	46	35	6
Verschuren et al. (1999a, b)	-	-	9	2
Verschuren et al. (2000)	-	-	8	-
Cocquyt and De Wever (2002)	39	12	-	6
Stoof-Leichsenring et al. (2011, 2012)	40	-	-	-
Owino et al. (2020)	23	-	-	-
Total number of diatom taxa	123	132	43	15

## ﻿Results and discussion

Over the last decades, from the earliest start of diatom investigation of the Lake Naivasha system up to now, a total of 205 different species and infraspecific taxa have been reported: 132 from the main basin of Lake Naivasha, 123 from Crescent Island Crater, 43 from Oloidien and 15 from Sonachi (Table 2). When including unpublished studies of sediment core material this number increases to 310 (236, 149, 43 and 52 respectively) distributed over 66 genera. *Cymatopleura* and *Rhopalodia* are kept as separate genera and the species are not included in *Surirella* and *Epithemia* respectively as recently recommended (Ruck et al. 2016a, b; Cocquyt et al. 2018). However, some of the reported taxa are unidentified and referred to as “aff.” (3), “cf.” (38) and “sp.” (14). Probably a number of these belong to already identified taxa and fall within the variability of a species, while others are potentially new to science and should be the subject of further taxonomic research.

Taking into consideration only the identified species and infraspecific taxa, 7 taxa (3.4%) are considered to be endemic to tropical Africa (Sub-Saharan Africa without southern Africa), besides 4 pantropical (2.0%) and 2 taxa restricted to the African continent (1.0%). This proportion is very small compared to other tropical lakes such as Lake Tanganyika where in the northern part up to 13.1% of the reported diatoms have a distribution restricted to tropical Africa (Cocquyt 2000). However, the number of tropical African, pantropical and African diatom taxa can increase as the unidentified taxa and those referred to as “aff.” and “cf.” potentially are taxa with a restricted distribution. Two other remarks should also be noted, namely that it is quite possible that material from Lake Naivasha and adjacent lakes was misidentified, as often European and North American diatom floras were used, and secondly, that diatom species, originally described from tropical Africa, have erroneously been reported from other tropical regions or from temperate regions in Europe and North America. Examples of this second possibility are several *Nitzschia* species, such as *N.accommodata*, *N.confinis*, *N.latens*, *N.spiculoides*, *N.subcommunis* and *N.tarda*, all described by Hustedt (1949) from the formerly Albert National Park (Belgian Congo), nowadays the Virunga National Park in the eastern part of the Democratic Republic of the Congo and the Volcanoes National Park in Rwanda.

Molecular analysis confirmed the morphological identification of 14 of the 49 species and infraspecific taxa observed in the sediment cores studied by Stoof-Leichsenring et al. (2012). This implies that slightly more than a quarter of the observed taxa are cosmopolitan. The genetically identified species had a higher internal similarity range than those that had been found in taxonomic studies on supposedly cosmopolitan species (Abarca et al. 2014). In addition, there are very few molecular data available of African taxa to serve as a reference library. The remaining three quarters of the reported taxa comprise species with restricted distribution, such as restricted to the tropics, to Africa, or to tropical Africa. This supports our hypothesis mentioned above that the number of tropical African, African and pantropical taxa must be higher than the number obtained by the results of the distribution of taxa present in this checklist.

In the overview below, taxa are listed according to the systematics of Round et al. (1990), with some adaptations to accommodate genera described after its publication. Although this classification is not the most recent one, and major changes have already occurred on higher taxonomic level (e.g. Adl et al. 2018), we believe it gives a clear and workable reference list of the diatom taxa known from Lake Naivasha, especially because many researchers who include diatoms in their research on Lake Naivasha are not taxonomists. The classification given here includes classes, orders, families and genera. Within each family, genera are arranged alphabetically and so are the species and infra-specific taxa within the genera. The authorities are compliant with the International Plant Names Index (2022). The most current used synonyms are given, as well as the synonyms used in the published papers. For each species the literature is cited where this taxon was mentioned for Lake Naivasha and its satellite lakes in the most important publications dealing with diatom taxonomy. With regards to our own unpublished observations, reference is made to the sediment core in which the species was observed.

## ﻿Checklist

### ﻿Class Coscinodiscophyceae Round & R.M.Crawford, 1990

**Order Thalassiosirales Glezer & Makarova, 1986**

#### ﻿Family Thalassiosiraceae M.Lebour, 1930

##### Genus *Thalassiosira* Cleve, 1973

**1. *Thalassiosirafaurii* (Gasse) Hasle, 1978: 282, figs 61–69.**

*Coscinodiscusfaurii* Gasse nom. inval., 1975: 24, pl. 32 figs 1, 2.

**Observation.** Main basin: Stoof-Leichsenring et al. (2012), Cocquyt and De Wever (2002), NM91.1-S, NM93.1-S.

Crescent Island Crater: Cocquyt and De Wever (2002), NC93, NC20.

Sonachi: NS93.2-F.

**Occurrence.** Epiphytic, sediment core.

**2. *Thalassiosirarudolfi* (H.Bachmann) Hasle, 1978: 279, figs 51–60, 65.**

*Coscinodiscusrudolfi* H.Bachmann, 1939: 135, fig. 7. The specific epithet is *rudolfi* and not *rudolfii* because the species is named after Lake Rudolf.

**Observation.** Main basin: NM91.1-S, NM93.1-S.

Crescent Island Crater: Richardson and Richardson (1972), Van der Meeren et al. (2019), NC20.

Oloidien: Verschuren et al. (1999b).

Sonachi: NS93.2-F.

**Occurrence.** Sediment core.

**Remark.**Rich (1932) reported a *Coscinodiscus* sp. from bottom mud in the main basin of Naivasha, probably one of the *Thalassiosira* taxa mentioned above. In NS93.2-F from Sonachi a valve fragment of *Thalassiosira* was observed.

### ﻿Class Coscinodiscophyceae Round & R.M.Crawford, 1990

**Order Thalassiosirales Glezer & Makarova, 1986**

#### ﻿Family Stephanodiscaceae Glezer & Makarova, 1986

##### Genus *Cyclotella* (Kützing) Brébisson, 1838

**3. *Cyclotellameneghiniana* Kützing, 1844: 50, pl. 30 fig. 68.**

**Observation.** Main basin: Gasse (1986), Stoof-Leichsenring et al. (2011), Stoof-Leichsenring et al. (2012), NM91.1-S, NM93.1-S.

Crescent Island Crater: Richardson and Richardson (1972), Gasse (1986), Cocquyt and De Wever (2002), NC93, NC20.

Oloidien: Verschuren et al. (1999b).

Sonachi: NS93.2-F.

**Occurrence.** Epiphytic, bottom mud, sediment core.

**4. *Cyclotella* sp.**

**Observation.** Main basin: NM91.1-S, NM93.1-S.

Crescent Island Crater: NC93.

**Occurrence.** Sediment core.

##### Genus *Cyclostephanos* Round 1987

**5. *Cyclostephanosdamasii* (Hustedt) Stoermer & Håkansson, 1988: 346.**

*Stephanodiscusdamasii* Hustedt, 1949: 57, pl. I figs 2–5.

**Observation.** Main basin: Cocquyt and De Wever (2002).

Crescent Island Crater: Richardson and Richardson (1972).

**Occurrence.** Epiphytic, sediment core.

**6. *Cyclostephanosinvisitatus* (M.H.Hohn & Hellerman) E.C.Theriot, Stoermer & Håkansson, 1988: 256, figs 18–24.**

*Stephanodiscusinvisitatus* M.H.Hohn & Hellerman, 1963: 325, pl. 1 fig. 7.

**Observation.** Crescent Island Crater: NC20.

**Occurrence.** Sediment core.

A small *Cyclostephanos* taxon was observed in the sediment core NM91.1-S taken from the main basin of Lake Naivasha, which may be identical to *Cyclostephanosinvisitatus*.

##### Genus *Discostella* Houk & Klee, 2004

**7. *Discostellapseudostelligera* (Hustedt) Houk & Klee, 2004: 223, figs 109, 110.**

*Cyclotellapseudostelligera* Hustedt, 1939: 581, figs 1, 2.

**Observation.** Crescent Island Crater: NC93.

**Occurrence.** Sediment core.

**8. *Discostellastelligera* (Cleve & Grunow) Houk & Klee, 2004: 208.**

Cyclotellameneghinianavar.stelligera Cleve & Grunow, 1881: 22, pl. 5, fig. 63a, c.

*Cyclotellastelligera* (Cleve & Grunow) Van Heurck, 1882: pl. XCIV figs 22–26.

**Observation.** Main basin: Stoof-Leichsenring et al. (2011), Stoof-Leichsenring et al. (2012), NM91.1-S, NM93.1-S.

Crescent Island Crater: Richardson and Richardson (1972), NC93, NC20.

**Occurrence.** Sediment core.

Cocquyt and De Wever (2002) reported a Cyclotellacf.stelligera on herbarium material from the main basin of Lake Naivasha.

##### Genus *Lindavia* (Schütt) De Toni & Forti, 1900

**9. *Lindaviaglomerata* (H.Bachmann) Adesalu & Julius, 2017: 170.**

*Cyclotellaglomerata* H.Bachmann, 1911: 131, figs 106–108.

**Observation.** Main basin: Gasse (1986), NM91.1-S, NM93.1-S.

**Occurrence.** Plankton, sediment core.

##### Genus *Pantocsekiella* K.T.Kiss & Ács, 2016

**10. Pantocsekiellacf.comensis (Grunow) K.T.Kiss & Ács, 2016: 65.**

*Cyclotellacomensis* Grunow, 1882: pl. 93 figs 16, 17.

**Observation.** Main basin: Cocquyt and De Wever (2002).

**Occurrence.** Epiphytic.

**11. *Pantocsekiellakuetzingiana* (Thwaites) K.T.Kiss & Ács, 2016: 67.**

*Cyclotellakuetzingiana* Thwaites, 1848: 169, pl. XI fig. D 1–5.

**Observation.** Main basin: Gasse (1986) (and varieties), Owino et al. (2020), NM91.1-S, NM93.1-S.

Crescent Island Crater: Richardson and Richardson (1972), NC20.

**Occurrence.** Plankton, bottom mud, sediment core.

**12. *Pantocsekiellaocellata* (Pantocsek) K.T.Kiss & Ács, 2016: 62.**

*Cyclotellaocellata* Pantocsek, 1901: 134, pl. IV fig. 318.

**Observation.** Main basin: Gasse (1986), Cocquyt and De Wever (2002), Stoof-Leichsenring et al. (2012), Owino et al. (2020), NM91.1-S, NM93.1-S.

Crescent Island Crater: Richardson and Richardson (1972), NC20, NC93.

Lake Sonachi: NS93.2-F.

**Occurrence.** Bottom mud, epiphytic, sediment core.

##### Genus *Stephanodiscus* Ehrenberg, 1845

**13. Stephanodiscuscf.agassizensis Håkansson & H.J.Kling, 1989: 283, 285, figs 56–59.**

**Observation.** Crescent Island Crater: core NC20.

**Occurrence.** Sediment core.

**14. *Stephanodiscusastraea* (Kützing) Grunow, 1880: 114 complex.**

*Cyclotellaastraea* Kützing, 1849: 19.

**Observation.** Main basin: Owino et al. (2020).

Crescent Island Crater: Richardson and Richardson (1972).

**Occurrence.** Sediment core.

**15. Stephanodiscuscf.hantzschii Grunow, 1880: 115, pl. VII fig. 131.**

**Observation.** Crescent Island Crater: Richardson and Richardson (1972).

Main basin: NS91.1-S.

Lake Sonachi: NS93.2-F.

**Occurrence.** Sediment core.

**16. Stephanodiscuscf.minutulus (Kützing) Cleve & Möller, 1882: 300.**

*Cyclotellaminutula* Kützing, 1844: 50.

**Observation.** Main basin: Cocquyt and De Wever (2002).

Crescent Island Crater: Richardson and Richardson (1972).

**Occurrence.** Epiphytic, sediment core.

**Remark.** Beside the above mentioned *Stephanodiscus* taxa, Owino et al. (2020) reported an unidentified species in a sediment core taken from the main basin of Lake Naivasha.

### ﻿Class Coscinodiscophyceae Round & R.M.Crawford, 1990

**Order Aulacoseirales R.M.Crawford, 1990**

#### ﻿Family Aulacoseiraceae R.M.Crawford, 1990

##### Genus *Aulacoseira* Thwaites, 1848

**17. *Aulacoseiraagassizii* (Ostenfeld) Simonsen, 1979: 56.**

*Melosiraagassizii* Ostenfeld, 1909: 179, pl. 2 figs 18–20.

**Observation.** Main basin: Gasse (1986), NM93.1-S.

Crescent Island Crater: Richardson and Richardson (1972), Gasse (1986), NC93, NC20.

**Occurrence.** Plankton, bottom mud, sediment core.

**18. *Aulacoseiraalpigena* (Grunow) Krammer, 1991: 93, figs 1–15.**

Melosiradistansvar.alpigena Grunow, 1882: pl. LXXXVI figs 28, 29.

Aulacoseiradistansvar.alpigena (Grunow) Simonsen, 1979: 57.

**Observation.** Main basin: NM93.1-S and cf. this taxon in NM91.1-S.

Crescent Island Crater: NC93.

**Occurrence.** Sediment core.

**19. *Aulacoseiraambigua* (Grunow) Simonsen, 1979: 56.**

Melosiracrenulatavar.ambigua Grunow, in Van Heruck 1882: pl. 88 figs 12–15.

*Melosiraambigua* (Grunow) O.Müller, 1903: 332.

**Observation.** Main basin: Rich (1932), Gasse (1986), Cocquyt and De Wever (2002), Stoof-Leichsenring et al. (2011), Stoof-Leichsenring et al. (2012), Owino et al. (2020), NM91.1-S, NM93.1-S.

Crescent Island Crater: Richardson and Richardson (1972), Van der Meeren et al. (2019), NC20, and cf. this taxon in NC93.

Lake Oloidien: Verschuren et al. (1999b), Verschuren et al. (2000b).

**Occurrence.** Plankton, epiphytic, bottom mud, sediment core.

**20. *Aulacoseiradistans* (Ehrenberg) Simonsen, 1979: 57.**

*Gaillonelladistans* Ehrenberg, 1836: 221, pl. III fig. 5.

*Melosiradistans* (Ehrenberg) Kützing, 1844: 54.

**Observation.** Main basin: Stoof-Leichsenring et al. (2011), Stoof-Leichsenring et al. (2012).

Crescent Island Crater: Richardson and Richardson (1972).

**Occurrence.** Sediment core.

**21. Aulacoseiradistansvar.africana (O.Müller) Simonsen, 1979: 57.**

Melosiradistansvar.africana O.Müller, 1904: 293, pl. IV figs 32, 33.

**Observation.** Main basin: Stoof-Leichsenring et al. (2011), NM91.1-S, NM93.1-S.

Crescent Island Crater: NC93, NC20.

**Occurrence.** Sediment core.

**22. Aulacoseiracf.goetzeana (O.Müller) Simonsen, 1979: 58.**

*Melosiragoetzeana* O.Müller, 1904: 290, pl. IV fig. 20.

**Observation.** Crescent Island Crater: NC20.

**Occurrence.** Sediment core.

**23. *Aulacoseiragranulata* (Ehrenberg) Simonsen, 1979: 58.**

*Gaillonellagranulata* Ehrenberg, 1843: 415.

*Melosiragranulata* (Ehrenberg) Ralfs in Pritchard, 1861: 820.

**Observation.** Main basin: Gasse (1986), Stoof-Leichsenring et al. (2011), Stoof-Leichsenring et al. (2012), NM91.1-S, NM93.1-S.

Crescent Island Crater: Richardson and Richardson (1972), Gasse (1986), NC93, NC20.

Lake Oloidien: Verschuren et al. (1999b), Verschuren et al. (2000b).

**Occurrence.** Plankton, bottom mud, sediment core.

Richardson and Richardson (1972) distinguish a variety of *A.granulata*, “*A.granulata* var. (coarse variety)”, which has much coarser areolae than the other valves of *A.granulata* and varieties observed in the studied sediment core materials. This taxon may be identified as Aulacoseiracf.goetzeana in core NC20.

**24. Aulacoseiragranulatavar.angustissima (O.Müller) Simonsen, 1979: 58.**

Melosiragranulatavar.angustissima O.Müller, 1899: 315.

**Observation.** Main basin: Gasse (1986), Stoof-Leichsenring et al. (2011), Stoof-Leichsenring et al. (2012), NM91.1-S, NM93.1-S.

Crescent Island Crater: Richardson and Richardson (1972), Gasse (1986), Van der Meeren et al. (2019), NC93, NC20.

**Occurrence.** Plankton, bottom mud, sediment core.

In NC93, the form curvata was distinguished within this taxon.

**25. *Aulacoseiraherzogii* (Lemmermann) Simonsen, 1979: 59.**

*Melosiraherzogii* Lemmermann, 1910: 316, figs 12, 13.

**Observation.** Crescent Island Crater: NC20.

**Occurrence.** Sediment core.

**26. *Aulacoseirahumilis* (A.Cleve) Genkal & Trifonova, in Trifonova and Genkal 2001: 315.**

Melosiradistansvar.humilis A.Cleve, 1939: 6, fig. 1.

Aulacoseiradistansvar.humilis (A.Cleve) Gasse, 1986: 76, pl. 3 figs 1–4, 7–9.

**Observation.** Crescent Island Crater: NC93.

**Occurrence.** Sediment core.

**27. *Aulacoseiraitalica* (Ehrenberg) Simonsen, 1979: 60.**

*Gaillonellaitalica* Ehrenberg, 1838: 171, pl. 10 fig. 6.

*Melosiraitalica* (Ehrenberg) Kutzing, 1844: 55, pl. 2 fig. 6.

**Observation.** Main basin: Bachmann (1938).

Crescent Island Crater: in NC93 a taxon was identified as A.cf.italica.

Lake Oloidien: Verschuren et al. (1999b), Verschuren et al. (2000b).

**Occurrence.** Phytoplankton, sediment core.

**28. Aulacoseiraitalicavar.bacilligera (O.Müller) Gasse, 1986: 81.**

Melosiraitalicavar.bacilligera O.Müller, 1844: 55, pl. 2 fig. 6.

**Observation.** Main basin: Cocquyt and De Wever (2002).

**Occurrence.** Epiphytic.

**29. *Aulacoseirajonensis* (Grunow) Houk & Klee, 2007: 99, pl. LXXXII figs 1–13, pl. LXXXIII figs 1–8.**

Melosiragranulatavar.jonensis Grunow, 1882: pl. LXXXVII figs 23–26.

Aulacoseiragranulatavar.jonensis (Grunow) Simonsen, 1979: 58.

**Observation.** Crescent Island Crater: Richardson and Richardson (1972), NC93.

**Occurrence.** Sediment core.

**30. *Aulacoseiramuzzanensis* (F.Meister) Krammer, 1991: 98.**

*Melosiramuzzanensis* F.Meister, 1912: 41, 232, pl. 1 fig. 10.

Aulacoseiragranulatavar.muzzanensis (F.Meister) Simonsen, 1979: 59.

**Observation.** Main basin: NM91.1-S, NM93.1-S.

Crescent Island Crater: cf. this taxon in NC20.

**Occurrence.** Sediment core.

**31. *Aulacoseiranyassensis* (O.Müller) Simonsen, 1979: 61.**

*Melosiranyassensis* O.Müller, 1904: 285, pl. III fig. 3.

*Melosiranyassensis* [subsp. devriesii] f. minor O.Müller, 1904: 3287, pl. III fig. 2.

**Observation.** Main basin: Rich (1932), Owino et al. (2020), NM91.1-S, NM93.1-S.

**Occurrence.** Phytoplankton, sediment core.

**32. Aulacoseiranyassensisvar.victoriae (O.Müller) Simonsen, 1979: 61.**

Melosiranyassensisvar.victoriae O.Müller, in Ostenfeld 1908: 338.

**Observation.** Crescent Island Crater: Richardson and Richardson (1972).

**Occurrence.** Sediment core.

According to AlgaeBase (Guiry and Guiry 2022) the taxonomic status of this taxon requires further investigation.

**33. *Aulacoseirapyxis* (O.Müller) Simonsen, 1979: 62.**

*Melosirapyxis* O.Müller, 1904: 291, pl. IV figs 23–5.

**Observation.** Main basin: Rich (1932).

**Occurrence.** Bottom mud.

**Remark.** Besides the seventeen above mentioned *Aulacoseira* taxa, an unidentified species was reported in the main basin by Stoof-Leichsenring et al. (2011), Stoof-Leichsenring et al. (2012), and in NM91.1-S, NM93.1-S and NS93.2-F. In addition, Owino et al. (2020) erroneously mentioned two species with the generic name of *Aulacoseira*: *A.schroidera* and *A.ulna*. The latter is probably *Ulnariaulna*. We have no idea which species is meant by the former but the most similar name is *Melosiraschroederi* (Wołoszyńska 1914: 186, pl. III figs 11, 12, 14) described from Lake Victoria.

### ﻿Class Coscinodiscophyceae Round & R.M.Crawford, 1990

**Order Chaetocerotales Round & R.M.Crawford, 1990**

#### ﻿Family Chaetocerotaceae Ralfs in Pritchard, 1861

##### Genus *Chaetoceros* Ehrenberg, 1844

**34. *Chaetoceros* sp.**

**Observation.** Main basin: Stoof-Leichsenring et al. (2011).

Lake Sonachi: NS93.2-F.

**Occurrence.** Sediment core.

### ﻿Class Fragilariophyceae, Round

**Order Fragilariales P.C.Silva, 1962**

#### ﻿Family Fragilariaceae Greville, 1833

##### Genus *Belonastrum* (Lemmermann) Round & Maidana, 2001

**35. *Belonastrumberolinense* (Lemmermann) Round & Maidana, 2001: 22.**

*Synedraberolinensis* Lemmermann, 1900: 31.

*Fragilariaberolinensis* (Lemmermann) Lange-Bertalot, 1993: 43, pl. 134 figs 21–25.

**Observation.** Crescent Island Crater: Richardson and Richardson (1972), NC20.

**Occurrence.** Sediment core.

##### Genus *Fragilaria* Lyngbye, 1819

**36. *Fragilariaamphicephaloides* Lange-Bertalot, 2013: 256, pl. 7 figs 7–10.**

*Synedraamphicephala* Kützing, 1844: 64, pl. 3 fig. 12.

*Fragilariaamphicephala* (Kützing) Lange-Bertalot nom. illeg., 2000.

**Observation.** Crescent Island Crater: NC20.

**Occurrence.** Sediment core.

**37. *Fragilariacapucina* Desmazières, 1830, no. 453.**

**Observation.** Main basin: Stoof-Leichsenring et al. 2012, Owino et al. 2020.

Lake Oloidien: Verschuren et al. (1999b), Verschuren et al. (2000b).

**Occurrence.** Sediment core.

**38. *Fragilariafragilarioides* (Grunow) Cholnoky, 1963: 169, pl. 25 figs 29, 30.**

Synedrarumpensvar.fragilarioides Grunow, 1881: pl. 40 fig. 12.

Fragilariarumpensvar.fragilarioides (Grunow) A.Cleve, 1953: 42, fig. 352b.

Fragilariacapucinavar.fragilarioides (Grunow) Ludwig & Flores, 1997: 58, figs 2–9.

**Observation.** Main basin: Cocquyt and De Wever (2002), NM91.1-S, NM93.1-S.

Crescent Island Crater: NC20.

**Occurrence.** Epiphytic, sediment core.

**39. *Fragilariaradians* (Kützing) D.M.Williams & Round, 1987: 269.**

*Synedraradians* Kützing, 1844: 64, pl. 14/7 figs 1–4.

Synedraacusvar.radians (Kützing) Hustedt, 1930: 155 fig. 171.

Synedraacusf.radians (Kützing) Hustedt, 1957: 237.

**Observation.** Main basin: Cocquyt and De Wever (2002), NM91.1-S, NM93.1-S.

Crescent Island Crater: Cocquyt and De Wever (2002), Van der Meeren et al. (2019), NC20.

**Occurrence.** Epiphytic, sediment core.

**40. *Fragilariarumpens* (Kützing) G.W.F.Carlson, 1913: 29.**

*Synedrarumpens* Kützing, 1844: 69, pl. 1 figs 4, 5.

*Fragilariacapucina* (Kützing) Lange-Bertalot ex Bukhtiyarova, 1995: 417.

**Observation.** Main basin: Gasse (1986), Stoof-Leichsenring et al. (2011), Stoof-Leichsenring et al. (2012).

Crescent Island Crater: Gasse (1986).

**Occurrence.** Plankton, bottom mud.

In NC20 an unspecified variety of this taxon was reported.

**41. Fragilariatabulatavar.truncata (Greville) Lange-Bertalot, 1938: 167, fig. 1 a–g.**

Echinellafasciculatavar.truncata Greville, 1832: pl. 16 fig. 4.

Synedravaucheriaevar.truncata (Greville) Rabenhorst, 1864: 132.

Fragilariavaucheriaevar.truncata (Greville) Stoermer & Yang, 2005: 1701.

**Observation.** Crescent Island Crater: NC20.

**Occurrence.** Sediment core.

**42. *Fragilariatenera* (W.Smith) Lange-Bertalot, 1980: 746.**

*Synedratenera* W.Smith, 1856: 98.

**Observation.** Main basin: Gasse (1986), NM91.1-S, NM93.1-S.

Crescent Island Crater: NC20.

**Occurrence.** Plankton, bottom mud, sediment core.

**43. *Fragilariavaucheriae* (Kützing) J.B.Petersen, 1938: 167, fig. 1 a–g.**

*Exilariavaucheriae* Kützing, 1833: 32, fig. 38.

*Synedravaucheriae* (Kützing) Kützing, 1844: 65, pl. 14 fig. 4.

Fragilariacapucinavar.vaucheriae (Kützing) Lange-Bertalot, 1980: 747.

**Observation.** Crescent Island Crater: NC20.

**Occurrence.** Sediment core.

A unidentified *Fragilaria* sp. was observed in the sediment core NM91.1-S taken from the main basin of Lake Naivasha. However, as the diatom analysis was performed at the beginning of the 1990s before the delineation of many now accepted genera within *Fragilaria* s.l., we cannot determine the current genus of this species.

##### Genus *Pseudostaurosira* D.M.Williams & Round, 1988

**44. *Pseudostaurosirabrevistriata* (Grunow) D.M.Williams & Round, 1988: 276, figs 28–31.**

*Fragilariabrevistriata* Grunow in Van Heurck, 1885: 157, pl. 45 fig. 32.

*Staurosirabrevistriata* (Grunow) Grunow, 1884: 101.

**Observation.** Crescent Island Crater: Richardson and Richardson (1972), NC20.

Lake Sonachi: NS93.2-F.

**Occurrence.** Sediment core.

##### Genus *Punctastriata* D.M.Williams & Round, 1988

**45. *Punctastriatalancettula* (Schumann) P.B.Hamilton & Siver, 2008: 363.**

*Fragilarialancettula* Schumann, 1867: 52, pl. 1 fig. 4.

Fragilariapinnatavar.lancettula (Schumann) Hustedt, 1913: pl. 297 figs 51, 59–64.

Staurosirellapinnatavar.lancettula (Schumann) Siver, 2005: 197.

**Observation.** Crescent Island Crater: Richardson and Richardson (1972), NC20.

**Occurrence.** Sediment core.

##### Genus *Staurosira* Ehrenberg, 1843

**46. *Staurosirabinodi* s (Ehrenberg) Lange-Bertalot, 2011: 260, pl. 10 figs 41–57.**

*Fragilariabinodis* Ehrenberg, 1854: 12, pl. V:II fig. 26, pl. VI:I fig. 43, pl. X fig. 15.

Fragilariaconstruensf.binodis (Ehrenberg) Hustedt, 1957: 231.

Staurosiraconstruensvar.binodis (Ehrenberg) P.B.Hamilton, 1992: 29.

Pseudostaurosiraconstruensvar.binodis (Ehrenberg) Edlund nom. inval., 1994: 12.

*Pseudostaurosirabinodis* (Ehrenberg) Edlund, 2001: 88.

**Observation.** Crescent Island Crater: Richardson and Richardson (1972).

**Occurrence.** Sediment core.

Richardson and Richardson (1972) mention Fragilariaconstruensvar.binodis (Ehrenberg) Grunow. We think that they wanted to cite Fragilariaconstruensf.binodis (Ehrenberg) Hustedt and have erroneously put Grunow as author instead of Hustedt. That they might have meant Fragilariaconstruensvar.binodis Stockmayer nom. inval. (Stockmayer 1909: 75) seems unlikely. Moreover, according to Guiry and Guiry (2022) “The taxonomic and/or nomenclatural status of this taxon is in some way unresolved and requires further investigation”.

**47. *Staurosiraconstruens* Ehrenberg, 1843: 424.**

*Fragilariaconstruens* (Ehrenberg) Grunow, 1862: 101.

Staurosiraventervar.construens (Ehrenberg) Cleve & Möller, 1879: 270–271.

**Observation.** Main basin: NM93.1-S, and cf. this taxon in NM91.1-S.

Crescent Island Crater: Richardson and Richardson (1972), NC20.

Lake Sonachi: NS93.2-F.

**Occurrence.** Sediment core.

**48. Staurosiraconstruensvar.exigua (W.Smith) H.Kobayasi, 2002: 90.**

*Triceratiumexiguum* W.Smith, 1856: 87.

Fragilariaconstruensvar.exigua (W.Smith) Schulz-Danzig, 1920: 750, figs 9–16.

*Fragilariaexigua* (W.Smith) Lemmermann nom. illeg., 1908: 409.

**Observation.** Crescent Island Crater: Richardson and Richardson (1972).

**Occurrence.** Sediment core.

**49. *Staurosiradubia* Grunow nom. inval. in Cleve & Möller, 1879: 270–271.**

Fragilarialeptostauronvar.dubia (Grunow) Hustedt nom. inval., 1931: 254, figs 668h–i.

**Observation.** Crescent Island Crater: Gasse (1986).

**Occurrence.** Bottom mud.

According to Guiry and Guiry (2022) the taxonomic or nomenclatural status (or both) of this entity is in some way unresolved and requires further investigation.

**50. *Staurosirainflata* (Heiden) A.Rusanov, Ács, E.Morales & Ector, 2018: 341, figs 3, 20–25, 30–43.**

*Synedrainflata* Heiden, 1900: 14, fig. 19.

*Fragilariaheidenii* Østrup, 1910: 190, pl. 5 fig. 118.

**Observation.** Main basin: core NM91.1-S, core NM93.1-S.

**Occurrence.** Sediment core.

**51. *Staurosiraleptostauron* (Ehrenberg) Kulikovskiy & Genkal, 2011: 363, pl. 2 figs. 1–6, pl. 8 fig. 1.**

*Biblariumleptostauron* Ehrenberg, 1854: 106, figs. 5–8.

*Staurosiraleptostauron* (Ehrenberg) D.M.Williams & Round, 1988: 276, figs. 22, 23.

**Observation.** Crescent Island Crater: NC20.

**Occurrence.** Sediment core.

**52. *Staurosirasubsalina* (Hustedt) Lange-Bertalot, 2004: 115.**

Fragilariaconstruensvar.subsalina Hustedt, 1925: 106, figs. 5–8.

Fragilariaconstruensf.subsalina (Hustedt) Hustedt, 1957.

*Pseudostaurosirasubsalina* (Hustedt) E.A.Morales, 2005: 115.

**Observation.** Crescent Island Crater: NC20.

Lake Sonachi: NS93.2-F.

**Occurrence.** Sediment core.

**53. *Staurosiraventer* (Ehrenberg) Cleve & J.D.Möller, 1879: 242.**

*Fragilariaventer* Ehrenberg, 1856: 87.

Fragilariaconstruensvar.venter (Ehrenberg) Grunow, in Van Heurck 1881: pl. 45 figs 21b, 23, 24b, 26a, 26b.

Fragilariaconstruensvar.venter (Ehrenberg) P.B.Hamilton, 1992: 29.

**Observation.** Crescent Island Crater: NC20.

**Occurrence.** Sediment core.

##### Genus *Staurosirella* D.M.Williams & Round, 1988

**54. *Staurosirellaafricana* (Hustedt) D.M.Williams & Round, 1988: 276.**

*Fragilariaafricana* Hustedt, 149: 62, pl. 2 figs 29–34.

**Observation.** Crescent Island Crater: NC20.

**Occurrence.** Sediment core.

**55. *Staurosirellapinnata* (Ehrenberg) D.M.Williams & Round, 1988: 274.**

*Fragilariapinnata* Ehrenberg, 1843: 415, pl. 3 figs 6, 8.

*Punctastriatapinnata* (Ehrenberg) D.M.Williams & Round, 1987: 278.

**Observation.** Main basin: Gasse (1986), NM91.1-S, NM93.1-S.

Crescent Island Crater: Richardson and Richardson (1972), NC20.

Lake Sonachi: NS93.2-F.

**Occurrence.** Plankton, sediment core.

In NC20 from Crescent Island, an unidentified variety of *Staurosirellapinnata* was reported.

**56. *Staurosirella* sp.**

**Observation.** Crescent Island Crater: NC20.

**Occurrence.** Sediment core.

##### Genus *Synedra* Ehrenberg, 1830

**57. *Synedracunningtonii* (Kützing) G.S.West, 1907: 151, pl. 8 fig. 4.**

**Observation.** Main basin: NM93.1-S, and cf. this taxon in NM91.1-S.

Crescent Island Crater: Richardson and Richardson (1972), NC20.

**Occurrence.** Sediment core.

The observed valves of this taxon in the materials of NM93.1-S. appear morphologically closely related to *Fragilariananana* and *F.tenera*, especially the nearly straight valves that are only slightly constricted/deformed mid-valve.

##### Genus *Ulnaria* (Kützing) Compère, 2001

**58. *Ulnariaacus* (Kützing) Aboal, 2003: 102.**

*Synedraacus* Kützing, 1844: 68, pl. 15 fig. VII.

Fragilariaulnavar.acus (Kützing) Lange-Bertalot, 1980: 745.

Fragilariaulnaf.acus (Kützing) Krammer & Lange-Bertalot ,1991: 144, fig: 122: 11–13, fig. 119: 8.

Ulnariaulnavar.acus (Kützing) Compère nom. inval., 2003: 70, pl. 12 figs 8–10.

**Observation.** Main basin: Gasse (1986), Cocquyt and De Wever (2002), Stoof-Leichsenring et al. (2011), Stoof-Leichsenring et al. (2012), Owino et al. (2020), NM91.1-S, NM93.1-S.

Crescent Island Crater: Richardson and Richardson (1972), Gasse (1986), Cocquyt and De Wever (2002), NC20.

Lake Oloidien: Gasse (1986).

**Occurrence.** Plankton, epiphytic, bottom mud, sediment core.

**59. *Ulnariaamphirhynchus* (Ehrenberg) Compère & Bukhtiyarova, 2006: 280.**

*Synedraamphirhynchus* Ehrenberg, 1843: 425, pl. III fig. I.25.

Synedraulnavar.amphirhynchus (Ehrenberg) Grunow, 1862: 397.

Ulnariaulnavar.amphirhynchus (Ehrenberg) Aboal, 2003: 113.

**Observation.** Crescent Island Crater: NC20.

**Occurrence.** Sediment core.

**60. *Ulnariabiceps* (Kützing) Compère, 2006: 281.**

*Synedrabiceps* Kützing, 1844: 66, pl. 14/18, 14/21 fig. 13.

Synedraulnaf.biceps (Kützing) Hustedt, 1957: 236.

Fragilariaulnavar.biceps (Kützing) Compère, 1991: 214.

*Fragilariabiceps* (Kützing) Lange-Bertalot nom. illeg., 1993: 43, pl. 121 figs 1–5.

**Observation.** Crescent Island Crater: NC20

**Occurrence.** Sediment core.

**61. *Ulnariacontracta* (Østrup) E.A.Morales & M.L.Vis, 2007: 125.**

Synedraulnavar.contracta Østrup, 1901: 281 fig. 247.

Fragilariaulnavar.contracta (Østrup) Main, 1988: 96.

**Observation.** Crescent Island Crater: NC20.

**Occurrence.** Sediment core.

**62. *Ulnariadanica* (Kützing) Compère & Bukhtiyarova, 2006: 281.**

*Synedradanica* Kützing, 1844: 66, pl. 14 fig. 13.

Fragilariaulnavar.danica (Kützing) Kalinsky, 1982: 125.

*Fragilariadanica* (Kützing) Lange-Bertalot, 1996: 54, pl. 7 fig. 1, pl. 109 figs 1, 1'.

Synedraulnavar.danica (Kützing) Van Heurck, 1885: 151.

Synedraulnaf.danica (Kützing) Hustedt, 1957: 151.

**Observation.** Main basin: Rich (1932).

Crescent Island Crater: NC20.

**Occurrence.** Bottom mud, sediment core.

**63. *Ulnariadelicatissima* (W.Smith) Aboal & P.C.Silva, 2004: 361.**

*Synedradelicatissima* W.Smith, 1853: 72, pl. 12 fig. 94.

Synedraacusvar.delicatissima (W.Smith) Rabenhorst, 1864: 136.

*Fragilariadelicatissima* (W.Smith) Lange-Bertalot nom. illeg., 1980: 746.

**Observation.** Main basin: Bachmann (1938), Stoof-Leichsenring et al. (2011), Stoof-Leichsenring et al. (2012).

**Occurrence.** Plankton, sediment core.

**64. Ulnariadelicatissimavar.angustissima (Grunow) Aboal & P.C.Silva, 2004: 361.**

Synedradelicatissimavar.angustissima Grunow, 1881: pl. XXXIX fig. 10.

Synedraacusvar.delicatissima (Grunow) Van Heurck, 1885: 151.

**Observation.** Main basin: Stoof-Leichsenring et al. (2011), Stoof-Leichsenring et al. (2012).

**Occurrence.** Sediment core.

**65. *Ulnarianyansae* (G.S.West) D.M.Williams, 2007: 125.**

*Synedranyansae* G.S.West, 1907: 149, pl. 8 fig. 3.

**Observation.** Main basin: Owino et al. (2020).

**Occurrence.** Sediment core.

**66. *Ulnariaulna* (Nitzsch) Compère, 2001: 100.**

*Bacillariaulna* Nitzsch, 1817: 99, pl. V figs 1–10.

*Synedraulna* (Nitzsch) Ehrenberg, 1832: 87.

*Fragilariaulna* (Nitzsch) Lange-Bertaot, 1980: 745.

**Observation.** Main basin: Rich (1932), Gasse (1986), Stoof-Leichsenring et al. (2011), Stoof-Leichsenring et al. (2012), Owino et al. (2020), NM93.1-S, and cf. this taxon in NM91.1-S.

Crescent Island Crater: Richardson and Richardson (1972), Gasse (1986), NC20.

Lake Oloidien: Verschuren et al. (1999b), Verschuren et al. (2000b).

**Occurrence.** Plankton, bottom mud, sediment core.

**67. Ulnariaulnavar.spathulifera (Grunow) Aboal, 2003: 114.**

*Synedraspathulifera* Grunow, 1881: pl. XXXVIII fig. 4.

Synedraulnavar.spathulifera (Grunow) Van Heurck, 1885: 151, pl. 38 fig. 4.

Synedraulnaf.spathulifera (Grunow) Skabichevskij, 1960: 242, fig. 81.

Fragilariaulnavar.spathulifera (Grunow) Main, 1988: 96.

**Observation.** Crescent Island Crater: Richardson and Richardson (1972).

**Occurrence.** Sediment core.

### ﻿Class Bacillariophyceae Haeckel, 1878

**Order Eunotiales P.C.Silva, 1962**

#### ﻿Family Eunotiaceae Kützing, 1844

##### Genus *Eunotia* Ehrenberg, 1837

**68. *Eunotiabilunaris* (Ehrenberg) Schaarschmidt, 1880: 159.**

*Synedrabilunaris* Ehrenberg, 1832: 87.

Eunotialunarisvar.bilunaris (Ehrenberg) Grunow, 1885: pl. XXXV fig. 6B.

**Observation.** Crescent Island Crater: core NC20.

**Occurrence.** Sediment core.

**69. *Eunotiacurvata* (Kützing) Lagerstedt, 1884: 61.**

*Exilariacruvata* Kützing, 1833: No 112.

**Observation.** Main basin: Cocquyt and DeWever (2002), NM91.1-S.

Crescent Island Crater: Gasse (1986).

**Occurrence.** Plankton, epiphytic.

**70. Eunotiacf.incisa W.Smith ex W.Gregory**, **1854: 25, pl. IV fig. 4.**

Eunotiasudeticavar.incisa (W.Smith ex W.Gregory) Manguin, 1950.

**Observation.** Crescent Island Crater: NC20.

**Occurrence.** Sediment core.

**71. *Eunotiaminor* (Kützing) Grunow, 1881: pl. 33 figs 20, 21.**

Eunotiapectinalisvar.minor (Kützing) Rabenhorst, 1864: 74.

Eunotiapectinalisf.minor (Kützing) O.Müller, 1910: 117.

**Observation.** Main basin: Cocquyt and De Wever (2002), NM91.1-S, NM93.1-S.

Crescent Island Crater: Gasse (1986), NC20.

**Occurrence.** Plankton, epiphytic, sediment core.

**72. *Eunotiamonodon* Ehrenberg, 1843: 414, pl. 1 fig. V.7.**

**Observation.** Main basin: Gasse (1986).

**Occurrence.** Plankton, bottom mud, sediment core.

**73. *Eunotiapectinalis* (Kützing) Rabenhorst, 1864: 73.**

Eunotiapectinalisvar.stricta (Rabenhorst) Van Heurck, 1881: pl. 33 fig. 18.

Eunotiapectinalisf.elongata Grunow, 1881: pl. 33 fig. 16 (text 1885: 143).

Eunotiapectinalisvar.impressa O.Müller, 1898: 12.

*Eunotiapectinalis* [var. minus] f. impressa (O.Müller) Ant.Mayer, 1913: 63.

**Observation.** Main basin: Gasse (1986), Stoof-Leichsenring et al. (2012).

Crescent Island Crater: Gasse (1986).

**Occurrence.** Plankton, bottom mud, sediment core.

In core NC20 from Crescent Island, a Eunotiacf.pectinalis was observed.

**74. Eunotiacf.tenella (Grunow) Hustedt, 1913: 287, figs 20–25.**

Eunotiaarcusvar.tenella Grunow, 1881: 34, figs 5–6.

**Observation.** Crescent Island Crater: Richardson and Richardson (1972).

**Occurrence.** Sediment core.

**Remark.** Besides the seven above mentioned *Eunotia* taxa, an unidentified species was reported in the Crescent Island core NC20, and in the main basin core NM91.1-S and core NM93.1-S.

### ﻿Class Bacillariophyceae Haeckel, 1878

**Order Mastogloiales D.G.Mann, 1990**

#### ﻿Family Mastogloiaceae Mereschkowsky, 1903

##### Genus *Mastogloia* Thwaites ex W.Smith, 1856

**75. *Mastogloiaelliptica* (C.Agardh) Cleve, 1893: pl. 185 figs 24–27.**

Mastogloiadanseivar.elliptica (C.Agardh) Grunow, 1880: pl.4 fig. 19.

**Observation.** Crescent Island Crater: Richardson and Richardson (1972), NC20.

**Occurrence.** Sediment core.

**76. *Mastogloia* sp.**

**Observation.** Lake Sonachi: core NS 92-2-F.

**Occurrence.** Sediment core.

### ﻿Class Bacillariophyceae Haeckel, 1878

**Order Cymbellales D.G.Mann, 1990**

#### ﻿Family Rhoicospheniaceae Chen & Zhu, 1983

##### Genus *Rhoicosphenia* Grunow, 1860

**77. *Rhoicospheniaabbreviata* (C.Agardh) Lange-Bertalot, 1980: 586, pl. 1A, 3C, D fig. 5A.**

*Rhoicospheniacurvata* (Kützing) Grunow, 1860: 511.

**Observation.** Main basin: Gasse (1986).

Crescent Island Crater: Richardson and Richardson (1972), NC20.

**Occurrence.** Bottom mud, sediment core.

### ﻿Class Bacillariophyceae Haeckel, 1878

**Order Cymbellales D.G.Mann, 1990**

#### ﻿Family Anomoeoneidaceae D.G.Mann, 1990

##### Genus *Anomoeoneis* Pfitzer, 1871

**78. *Anomoeoneiscostata* (Kützing) Hustedt, 1959: 744, fig. 1111.**

Anomoeoneissphaerophoraf.costata (Kützing) A.M.Schmid, 1977: 321, 329; invalid.

**Observation.** Lake Oloidien: Gasse (1986).

Lake Sonachi: NS93.2-F.

**Occurrence.** Bottom mud, sediment core.

**79. *Anomoeoneissculpta* (Ehrenberg) Cleve, 1895: 6.**

Anomoeoneissphaerophoravar.sculpta (Ehrenberg) O.Müller, 1900: 303.

Anomoeoneissphaerophoraf.sculpta (Ehrenberg) Krammer, 1985: 13.

**Observation.** Main basin: Gasse 1986, core NM91.1-S.

Lake Oloidien: Gasse (1986).

Lake Sonachi: NS93.2-F.

**Occurrence.** Bottom mud, sediment core.

**80. *Anomoeoneissphaerophora* Pfitzer, 1871: 77, pl. 3 fig. 10.**

**Observation.** Main basin: Rich (1932), Gasse (1986), Cocquyt and De Wever (2002), Stoof-Leichsenring et al. (2012).

Crescent Island Crater: Richardson and Richardson (1972), NC20.

Lake Oloidien: Gasse (1986).

Lake Sonachi: NS93.2-F.

**Occurrence.** Epiphytic, bottom mud, sediment core.

Rich (1932) reported various forms of this taxon resembling A.sphaerophoraf.rostrata O.Müller, 1900: 303, pl. XII figs. 3–9.

**81. Anomoeoneissphaerophoravar.guentheri , 1900: 302, pl. 12 figs 6–9.**

Anomoeoneisvar.guentheri (O.Müller) A.Cleve, 1953: 202, fig. 1927f, invalid.

**Observation.** Main basin: Gasse (1986).

Lake Oloidien: Gasse (1986).

**Occurrence.** Bottom mud.

### ﻿Class Bacillariophyceae Haeckel, 1878

**Order Cymbellales D.G.Mann, 1990**

#### ﻿Family Cymbellaceae Greville, 1833

##### Genus *Cymbella* C.Agardh, 1830

**82. *Cymbellacistula* (Ehrenberg) O.Kirchner, 1878: 189.**

**Observation.** Main basin: Gasse (1986), Owino et al. (2020).

**Occurrence.** Plankton, sediment core.

Richardson and Richardson (1972) reported a Cymbellacf.cistula from sediment core material of Crescent Island, while, Gasse (1986) mentioned this taxon as *Cymbellacistula* (Hemprich) Grunow.

**83. Cymbellacistulavar.africana Cholnoky, 1958: 105, pl. 1 figs 14–16.**

**Observation.** Crescent Island Crater: core NC20.

**Occurrence.** Sediment core.

**84. *Cymbellacymbiformis* C.Agardh, 1830: 10.**

**Observation.** Main basin: Gasse (1986).

**Occurrence.** Plankton.

Gasse (1986) reported this taxon as *Cymbellacymbiformis* (Kützing) Hustedt.

**85. *Cymbellakappii* (Cholnoky) Cholnoky, 1956: 61, figs 17–20.**

Cymbellaturgidulavar.kappii Cholnoky, 1953: 142, figs 12–16.

**Observation.** Crescent Island Crater: NC20.

**Occurrence.** Sediment core.

**86. *Cymbellalanceolata* C.Agardh, 1830: 9.**

*Naviculalanceolata* (C.Agardh) Kützing, nom. illeg., 1844: 94, pl. 28 fig. 38, pl. 30 fig. 48.

**Observation.** Main basin: Rich (1932).

**Occurrence.** Bottom mud.

**87. *Cymbellasimonsenii* Krammer, 1985: 33, pl. 7 figs 1–9.**

**Observation.** Crescent Island Crater: NC20.

**Occurrence.** Sediment core.

**Remark.** A *Cymbella* sp. was reported by Richardson and Richardson (1972), Owino et al. (2020) and Cocquyt and De Wever (2002), while Stoof-Leichsenring et al. (2012) mentioned several *Cymbella* sp. Also in core NM91.1-S, a *Cymbella* sp. was observed. However, we cannot verify if these concern *Cymbella* s.s. or *Cymbella* s.l.

##### Genus *Cymbopleura* (Krammer) Krammer, 1999

**88. *Cymbopleuraamphicephala* (Nägeli ex Kützing) Krammer, 2003: 70, pl. 91 figs 1–18, pl. 93 figs 2–8.**

*Cymbellaamphicephala* Nägeli ex Kützing, 1849: 890.

**Observation.** Main basin: Gasse (1986).

**Occurrence.** Plankton.

##### Genus *Encyonema* Kützing, 1834

**89. *Encyonemaelginense* (Krammer) D.G.Mann, 1990: 666.**

*Cymbellaelginensis* Krammer, 1980: 136, fig. 23.

*Cymbellaturgida* W.Gregory, 1856: 5, pl. 1 fig. 18.

*Encyonematurgidum* (W.Gregory) Grunow, 1875: pl. 10 figs 49–53.

**Observation.** Main basin: Gasse (1986).

Crescent Island Crater: Gasse (1986).

Lake Oloidien: Gasse (1986).

**Occurrence.** Plankton, bottom mud.

**90. *Encyonemagracile* Rabenhorst, 1853: 25, pl. 10.**

*Cymbellagracilis* (Rabenhorst) Cleve, 1894: 169.

**Observation.** Main basin: Rich (1932).

**Occurrence.** Bottom mud.

**91. *Encyonemamesianum* (Cholnoky) D.G.Mann, 1990: 666.**

*Cymbellamesiana* Cholnoky, 1955: 160, figs 11, 12.

**Observation.** Crescent Island Crater: NC93, NC20.

**Occurrence.** Sediment core.

**92. Encyonemacf.minutum (Hilse) D.G.Mann, 1990: 667.**

*Cymbellaminuta* Hilse, 1862: No. 1261.

Cymbellaventricosavar.minuta (Hilse) A.Cleve, 1955: 125, figs 1177g–i.

**Observation.** Main basin: NM91.1-S, NM93.1-S.

**Occurrence.** Sediment core.

**93. *Encyonemamuelleri* (Hustedt) D.G.Mann, 1990: 667.**

*Cymbellamuelleri* Hustedt, 1937: 425.

*Cymbellamuelleri* Cholnoky nom. inval., 1953: 141.

Cymbellagrossestriatavar.obtusiuscula O.Müller, 1905: 154, pl. 1 fig. 13.

**Observation.** Main basin: Rich (1932), Gasse (1986), Cocquyt and De Wever (2002), Stoof-Leichsenring et al. (2011), Stoof-Leichsenring et al. (2012), NM91.1-S, NM93.1-S.

Crescent Island Crater: Richardson and Richardson (1972), Cocquyt and De Wever (2002), NC20.

Lake Oloidien: Gasse (1986).

Lake Sonachi: Cocquyt and De Wever (2002).

**Occurrence.** Plankton, epiphytic, bottom mud, sediment core.

**94. *Encyonemaneomesianum* Krammer, 1997: 5, pl. 191 figs 7–9.**

Cymbellaminutavar.pseudogracilis (Cholnoky) Reimer, 1975: 50, pl. 9 figs 1a–2b.

Encyonemaminutumvar.pseudogracile (Cholnoky) Czarnecki, 1994: 157.

Cymbellaturgidavar.pseudogracilis Cholnoky, 1958: 112; pl. 2 figs 49–50.

**Observation.** Crescent Island Crater: Richardson and Richardson (1972).

**Occurrence.** Sediment core.

**95. *Encyonemaneomuelleri* Krammer, 1997: 142, figs 23–27.**

**Observation.** Crescent Island Crater: NC20.

**Occurrence.** Sediment core.

**96. *Encyonemasilesiacum* (Bleisch) D.G.Mann, 1990: 667.**

*Cymbellasilesiaca* Bleisch, 1864: No. 1802.

Cymbellaminutavar.silesiaca (Bleisch) Reimer, 1975: 49, pl. 8 figs 7a–10b.

Cymbellaventricosavar.silesiaca (Bleisch) A.Cleve, 1955: 124, figs 1177d–f.

**Observation.** Crescent Island Crater: NC93, NC20.

Main basin: Cocquyt and De Wever (2002), NM91.1-S, NM93.1-S.

**Occurrence.** Epiphytic, sediment core.

**97. *Encyonemaventricosum* (C.Agardh) Grunow, 1875: pl. 10 fig. 59.**

*Cymbellaventricosa* (C.Agardh) C.Agardh, 1830: 9.

**Observation.** Crescent Island Crater: Gasse (1986).

Main basin: Gasse (1986).

Lake Oloidien: Gasse (1986).

**Occurrence.** Plankton, littoral, bottom mud.

Bachmann (1938) reported *Cymbellaventricosa* Kützing from the littoral zone of Lake Naivasha. According to Guiry and Guiry (2022)*Cymbellaventricosa* Kützing, 1844 is a nom. illeg. and the taxonomic or nomenclatural status (or both) of this entity is in some way unresolved and requires further investigation. Probably the taxon Bachmann observed is the same species as *Encyonemaventricosum* reported by Gasse (1986).

**98. *Encyonema* sp.**

**Observation.** Crescent Island Crater: NC20.

**Occurrence.** Sediment core.

##### Genus *Encyonopsis* Krammer, 1997

**99. *Encyonopsismicrocephala* (Grunow) Krammer**, 1997: 91.

*Cymbellamicrocephala* Grunow, 1885: 63.

**Observation.** Crescent Island Crater: NC20.

**Occurrence.** Sediment core.

**100. *Encyonopsis* sp.**

**Observation.** Crescent Island Crater: NC20.

**Occurrence.** Sediment core.

##### Genus *Geissleria* Lange-Bertalot & Metzeltin, 1996

**101. Geissleriacf.acceptata (Hustedt) Lange-Bertalot & Metzeltin, 1996: 64.**

*Naviculaacceptata* Hustedt, 1950: 398, pl. 38 figs 66, 67.

**Observation.** Crescent Island Crater: NC20.

**Occurrence.** Sediment core.

**102. Geissleriacf.decussis (Østrup) Lange-Bertalot & Metzeltin, 1996: 65, pl. 104 fig. 2, pl. 125 figs 3–6.**

*Naviculadecussis* Østrup, 1910: 77, pl. 2 fig. 50.

*Navigeiadecussis* (Østrup) Bukhtiyarova, 2013: 168.

**Observation.** Crescent Island Crater: NC20.

**Occurrence.** Sediment core.

**103. *Geissleriapseudolagerstedtii* (Cholnoky) J.C.Taylor & Cocquyt, 2019: 1.**

*Naviculapseudolagerstedtii* Cholnoky, 1960: 75, pl. 6 fig. 326.

**Observation.** Crescent Island Crater: Richardson and Richardson (1972).

**Occurrence.** Sediment core.

**104. *Geissleria* sp.**

**Observation.** Crescent Island Crater: NC20.

**Occurrence.** Sediment core.

##### Genus *Placoneis* Mereschkowsky, 1903

**105. Placoneiscf.dicephala (Ehrenberg) Mereschkowsky, 1903: 7, pl. 1 figs 11–13, 21, 22.**

*Naviculadicephala* Ehrenberg, 1838: 185.

**Observation.** Crescent Island Crater: NC20.

**Occurrence.** Sediment core.

**106. Placoneiscf.gastrum (Ehrenberg) Mereschkowsky, 1903: 13, pl. 1 fig. 17.**

*Naviculagastrum* (Ehrenberg) Kutzing, 1844: 94, pl. 28 fig. 56c.

**Observation.** Crescent Island Crater: NC20.

**Occurrence.** Sediment core.

**107. *Placoneishambergii* (Hustedt) Bruder, 2007: 349.**

*Naviculahambergii* Hustedt, 1924: 562, pl. 17 fig. 2.

**Observation.** Crescent Island Crater: NC20.

**Occurrence.** Sediment core.

### ﻿Class Bacillariophyceae Haeckel, 1878

**Order Cymbellales D.G.Mann, 1990**

#### ﻿Family Gomphonemataceae Kützing, 1844

##### Genus *Gomphonema* Ehrenberg, 1832

**108. *Gomphonemaacuminatum* Ehrenberg, 1832: 88.**

**Observation.** Main basin: Owino et al. (2020).

**Occurrence.** Sediment core.

**109. Gomphonemaacuminatumvar.longiceps (Ehrenberg) N.Abarca & R.Jahn, 2020: 36.**

*Gomphonemalongiceps* Ehrenberg, 1854: pl. 10/1 fig. 21.

**Observation.** Crescent Island Crater: Richardson and Richardson (1972).

**Occurrence.** Sediment core.

**110. *Gomphonemaaffine* Kützing, 1844: 86, pl. 30 fig. 54.**

Gomphonemalanceolatumvar.affine (Kützing) A.Cleve, 1932: fig. 254c.

**Observation.** Crescent Island Crater: NC20.

**Occurrence.** Sediment core.

**111. Gomphonemacf.angustatum (Kützing) Rabenhorst, 1864: 283.**

**Observation.** Crescent Island Crater: NC20.

**Occurrence.** Sediment core.

**112. *Gomphonemaaugur* Ehrenberg, 1841: 211.**

**Observation.** Crescent Island Crater: NC20.

**Occurrence.** Sediment core.

**113. *Gomphonemagracile* Ehrenberg, 1838: 217, pl. 18 fig. 3.**

**Observation.** Main basin: Gasse (1986), Cocquyt and De Wever (2002), Stoof-Leichsenring et al. (2011), Stoof-Leichsenring et al. (2012), NM91.1-S.

Crescent Island Crater: Richardson and Richardson (1972), Gasse (1986), Cocquyt and De Wever (2002), NC20.

Lake Oloidien: Gasse (1986), Verschuren et al. (2000b).

Lake Sonachi: Verschuren et al. (1999a), Cocquyt and De Wever (2002), NS93.2-F.

**Occurrence.** Plankton, epiphytic, bottom mud, sediment core.

**114. Gomphonemagracilef.turris Hustedt, 1937: 439, pl. 28 figs 14–16.**

**Observation.** Main basin: Cocquyt and De Wever (2002).

**Occurrence.** Epiphytic.

**115. Gomphonemacf.insigne W.Gregory, 1856: 12, pl. 1 fig. 39.**

**Observation.** Crescent Island Crater: NC20.

**Occurrence.** Sediment core.

**116. *Gomphonemaintricatum* Kützing, 1844: 87, pl. 9 fig. 4.**

**Observation.** Main basin: Rich (1932), Gasse (1986), Stoof-Leichsenring et al. (2012), NM91.1-S, NM93.1-S.

Crescent Island Crater: Gasse (1986), NC20.

**Occurrence.** Plankton, bottom mud, sediment core.

Richardson and Richardson (1972) reported Gomphonemacf.intricatum from sediment core material of Crescent Island.

**117. *Gomphonemalanceolatum* Ehrenberg nom. illeg., 1843: 306, pl. II fig. 37.**

**Observation.** Main basin: Rich (1932), Gasse (1986), Stoof-Leichsenring et al. (2012), NM91.1-S, NM93.1-S.

Crescent Island Crater: Richardson and Richardson (1972), Gasse (1986), NC20.

Lake Oloidien: Gasse (1986).

**Occurrence.** Plankton, bottom mud, sediment core.

*Gomphonemalanceolatum* has long been a very common name of a diatom taxon by which various *Gomphonema* species and multiple forms of *Gomphonemaaffine* were referred to (Reichardt 1999). *Gomphonemaaffine* is a common species of tropical and sub-tropical regions and sometimes frequently found (Reichardt 1999). For this reason, in this paper we have not put the taxon observed in Lake Naivasha and its satellite lakes in synonymy with *Gomphonemagrunowii* R.M.Patrick & Reimer (1975: 131, pl. 17 fig. 6) as mentioned by Guiry in Guiry and Guiry (2022).

**118. *Gomphonemanaviculoides* W.Smith, 1856: 98.**

Naviculagracilevar.naviculoides (W.Smith) Grunow, 1880: pl. 24 fig. 13.

**Observation.** Main basin: Bachmann (1938).

**Occurrence.** Littoral.

**119. *Gomphonemaparvulum* (Kützing) Kützing, 1849: 65.**

*Gomphonemaclavatum* Ehrenberg, 1832: 88.

**Observation.** Main basin: Bachmann (1938), Gasse (1986), Stoof-Leichsenring et al. (2011), Stoof-Leichsenring et al. (2012), NM91.1-S, NM93.1-S.

Crescent Island Crater: Richardson and Richardson (1972), Gasse (1986), NC20.

Lake Oloidien: Gasse (1986).

Lake Sonachi: NS93.2-F.

**Occurrence.** Plankton, littoral, bottom mud, sediment core.

Cocquyt and De Wever (2002) reported Gomphonemacf.parvulum from epiphytic materials taken in Crescent Island, the main basin of Lake Naivasha and Lake Sonachi.

**120. *Gomphonemapseudoaugur* Lange-Bertalot, 1979: 202, figs 11–165.**

**Observation.** Main basin: Gasse (1986), Stoof-Leichsenring et al. (2011), Stoof-Leichsenring et al. (2012), NM93.1-S.

Crescent Island Crater: NC20.

Lake Oloidien: Gasse (1986).

**Occurrence.** Plankton, bottom mud, sediment core.

Cocquyt and De Wever (2002) reported Gomphonemacf.pseudoaugur from epiphytic materials taken in the main basin of Lake Naivasha and Lake Sonachi.

**121. *Gomphonemapumilum* (Grunow) E.Reichardt & Lange-Bertalot, 1991: 528, pl. 6 figs 4–11.**

Gomphonemaintricatumf.pumilum Grunow, 1880: pl. 24 figs 35, 36.

Gomphonemaintricatumvar.pumilum A.Cleve, 1932: 99, fig. 252b.

**Observation.** Crescent Island Crater: Richardson and Richardson (1972), core NC20.

**Occurrence.** Sediment core.

Gasse (1986) reported Gomphonemacf.pumilum from bottom mud materials taken in the main basin of Lake Naivasha.

**122. *Gomphonemasubapicatum* F.E.Fritsch & M.F.Rich, 1929: 109, pl. 6 A, B.**

**Observation.** Main basin: Bachmann (1938).

**Occurrence.** Littoral.

Bachmann (1938) remarked that the observed valves in Lake Naivasha were smaller than the dimensions given for this taxon by Fritsch & Rich (Fritsch et al. 1929).

**123. *Gomphonemasubclavatum* (Grunow) Grunow, 1884: 98, pl. 1 (A) fig. 13.**

**Observation.** Main basin: Rich (1932), Bachmann (1938).

Lake Oloidien: Gasse (1986).

**Occurrence.** Littoral, bottom mud.

**124. Gomphonemasubtilevar.sagitta (Schumann) Grunow, 1880: pl. 23 fig. 27.**

*Gomphonemasagitta* Schumann, 1863: 187, pl. 9 fig. 29.

**Observation.** Lake Oloidien: Gasse (1986).

**Occurrence.** Bottom mud.

**125. *Gomphonemasundaense* E.Reichardt, 2005: 129, pl. 6 figs 1–8.**

Gomphonemacleveivar.javanicum Hustedt, 1937: 441, pl. XXVII figs 6–13.

**Observation.** Main basin: Gasse (1986).

**Occurrence.** Bottom mud.

**126. *Gomphonematurris* Ehrenberg, 1843: 416.**

Gomphonemaaugurvar.turris (Ehrenberg) Lange-Bertalot, 1985: 44, pl. 37 figs 1–7, pl. 38 figs 1–4, 8–12.

Gomphonemaacuminatumvar.turris (Ehrenberg) Wolle, 1890: pl. 28 fig. 25.

**Observation.** Main basin: Bachmann (1938).

Crescent Island Crater: NC20.

**Occurrence.** Littoral, sediment core.

**127. Gomphonemavibriovar.pulvinatum (Braun ex Rabenhorst) R.Ross, 1986: 608.**

*Gomphonemapulvinatum* Braun ex Rabenhorst, 1853: 58.

Gomphonemaintricatumvar.pulvinatum Braun ex Rabenhorst, 1853: 58.

**Observation.** Main basin: Cocquyt and De Wever (2002).

**Occurrence.** Epiphytic.

**Remark.** Beside the above mentioned *Gomphonema* taxa, an unidentified *Gomphonema* sp. was reported by Owino et al (2020), and some small unidentified *Gomphonema* valves were observed in cores NC20, NM91.1-S and NM93.1-S.

##### Genus *Gomphoneis* Cleve 1894

**128. *Gomphoneisclevei* (Fricke) Gil, 1989: 267, 271, pl. 1 figs 4–12, pl. 4 figs 1–4, pl. 5 figs 1–4.**

*Gomphonemaclevei* Fricke, 1902: pl. 234 figs 44–46.

**Observation.** Main basin: NM93.1-S, and cf. this taxon in NM91.1-S.

Crescent Island Crater: NC20.

**Occurrence.** Sediment core.

### ﻿Class Bacillariophyceae Haeckel, 1878

**Order Cymbellales D.G.Mann, 1990**

#### ﻿Family Cymbellales incertae sedis

##### Genus *Gomphonella* Rabenhorst, 1853

**129. *Gomphonellaolivacea* (Hornemann) Rabenhorst, 1853: 61, pl. IX fig. 1.**

*Gomphonemaolivaceum* (Hornemann) Ehrenberg, 1838: 218.

**Observation.** Crescent Island Crater: NC20.

**Occurrence.** Sediment core.

### ﻿Class Bacillariophyceae Haeckel, 1878

**Order Achnanthales P.C.Silva ,1962**

#### ﻿Family Cocconeidaceae Kützing, 1844

##### Genus *Cocconeis* Ehrenberg, 1836

**130. *Cocconeisdisculus* (Schumann) Cleve, 1882: 139.**

*Naviculadisculus* Schumann, 1862: 21, fig. 23.

Naviculascutelloidesvar.disculus (Schumann) Torka, 1906: 15, fig. 3a.

**Observation.** Crescent Island Crater: Richardson and Richardson (1972).

**Occurrence.** Sediment core.

**131. Cocconeiscf.lineata Ehrenberg, 1849: 301, pl. 5 (part 2) fig. 44.**

Cocconeisplacentulavar.lineata (Ehrenberg) Van Heurck, 1885: 133 [Atlas pl. 30 figs 31, 32].

Cocconeisplacentulaf.lineata (Ehrenberg) Hustedt, 1957: 244.

**Observation.** Crescent Island Crater: NC93, NC20.

**Occurrence.** Sediment core.

Gasse (1986) mentioned that Cocconeisplacentulavar.lineata was associated with *Cocconeisplacentula* in the samples studied from East Africa. However, we cannot know if this taxon was observed by Gasse (1986) in Lake Naivasha and its satellites lakes.

**132. *Cocconeisplacentula* Ehrenberg, 1838: 194.**

Naviculapediculusvar.placentula (Ehrenberg) Grunow, 1867: 15.

**Observation.** Main basin: Gasse (1986), Cocquyt and De Wever (2002), Stoof-Leichsenring et al. (2012), NM91.1-S, NM93.1-S.

Crescent Island Crater: Richardson and Richardson (1972), Gasse (1986), Cocquyt and De Wever (2002), NC93, NC20

Lake Sonachi: Gasse (1986), Cocquyt and De Wever (2002).

**Occurrence.** Plankton, bottom mud, sediment core.

**133. Cocconeisplacentulavar.euglypta (Ehrenberg) Cleve, 1895: 170.**

*Cocconeiseuglypta* Ehrenberg, 1854: pl. XXXIV, pl. VI-A fig. 2.

Cocconeisplacentulaf.euglypta (Ehrenberg) Hustedt, 1957: 244.

**Observation.** Main basin: Rich 1932, NM91.1-S, NM93.1-S.

Crescent Island Crater: Richardson and Richardson (1972), NC93, NC20.

**Occurrence.** Bottom mud, sediment core.

Gasse (1986) mentioned that this variety was associated with the species in the samples studied from East Africa. However, as the variety *euglypta* is reported to be rare, we cannot know if this taxon was observed by Gasse (1986) in Lake Naivasha and its satellites lakes.

**134. *Cocconeis* sp.**

**Observation.** Crescent Island Crater: NC20.

**Occurrence.** Sediment core.

### ﻿Class Bacillariophyceae Haeckel, 1878

**Order Achnanthales P.C.Silva, 1962**

#### ﻿Family Achnanthidiaceae D.G.Mann, 1990

##### Genus *Achnanthes* Bory, 1822

**135. *Achnanthes* sp.**

**Observation.** Main basin: Cocquyt and De Wever (2002).

**Occurrence.** Epiphytic.

Cocquyt and De Wever (2002) reported a *Achnanthes* sp. epiphytic on *Nymphaeacaerulea* Savigny herbarium material collected in the main basin of Lake Naivasha. They only observed 3 valves of this taxon which was not illustrated.

The identity of the genus to which the taxon mentioned by Owino et al. (2020) as *Achnanthes* sp. belongs cannot be determined because these authors do not use the latest taxonomy in their paper.

##### Genus *Achnanthidium* Kützing, 1844

**136. Achnanthidiumcf.affine (Grunow) Czarnecki, 1994: 156.**

*Achnanthesaffinis* Grunow, 1880: 20.

Achnanthesminutissimavar.affinis (Grunow) Lange-Bertalot, 1989: 104.

**Observation.** Crescent Island Crater: NC20.

**Occurrence.** Sediment core.

**137. Achnanthidiumexiguumvar.heterovalvata (Krasske) Czarnecki, 1994: 157.**

Achnanthesexiguavar.heterovalvata Krasske, 1923: 193, figs 9a, 9b.

*Achnanthesheterovalvata* (Krasske) Frenguelli, 1942: 95, pl. 1 figs 9–10.

**Observation.** Main basin: Cocquyt and De Wever (2002).

Crescent Island Crater: Cocquyt and De Wever (2002).

Lake Sonachi: Cocquyt and De Wever (2002).

**Occurrence.** Epiphytic.

**138. *Achnanthidiumexile* (Kützing) Heiberg, 1863: 119.**

*Achnanthesexilis* Kützing, 1833: no. 12.

**Observation.** Crescent Island Crater: NC20.

**Occurrence.** Sediment core.

**139. *Achnanthidiumminutissimum* (Kützing) Czarnecki, 1994: 157.**

*Achnanthesminutissima* Kützing, 1833: 578, fig. 54.

Achnanthesminutissimavar.cryptocepala Grunow, 1880: pl. XXVII figs 41–44.

*Achnanthidiummicrocephalum* Kützing, 1844: 75, pl. XIII, XIX.

*Achnanthesmicrocephala* (Kützing) Grunow, 1880: 22.

*Achnanthescryptocephala* (Grunow) M.Peragallo, 1897: 4.

**Observation.** Main basin: Gasse (1986), Cocquyt and De Wever (2002), NM91.1-S, NM93.1-S.

Crescent Island Crater: NC20.

Lake Oloidien: Verschuren et al. (1999b), Verschuren et al. (2000b).

Lake Sonachi: Gasse (1986), NS93.2-F

**Occurrence.** Plankton, epiphytic, bottom mud, sediment core.

##### Genus *Gogorevia* Kulikovskiy, Glushchenko, Maltsev & Kociolek, 2020

**140. *Gogoreviaexilis* (Kützing) Kulikovskiy & Kociolek, 2020: 1610.**

*Achnanthesexigua* Grunow, 1880: 21.

*Achnanthidiumexiguum* (Grunow) Czarnecki, 1994: 157.

**Observation.** Crescent Island Crater: Richardson and Richardson (1972), NC20.

**Occurrence.** Sediment core.

##### Genus *Planothidum* Round & Bukhtiyarova, 1996

**141. *Planothidumrostratum* (Østrup) Lange-Bertalot, 1999: 279.**

*Achnanthesrostrata* Østrup, 1902: 35, pl. I fig. 11.

Achnantheslanceolatasubsp.rostrata (Østrup) Lange-Bertalot, 1991: 79.

Achnantheslanceolataf.rostrata (Østrup) Hustedt, 1957: 251.

**Observation.** Crescent Island Crater: Richardson and Richardson (1972).

**Occurrence.** Sediment core.

**142. *Planothidum* sp.**

**Observation.** Crescent Island Crater: NC20.

**Occurrence.** Sediment core.

##### Genus *Platessa* Lange-Bertalot, 2004

**143. Platessacf.strelnikovae M.D.Enache, M.Potapova & E.Morales, 2014: 240, figs 1–23.**

**Observation.** Crescent Island Crater: NC20.

**Occurrence.** Sediment core.

### ﻿Class Bacillariophyceae Haeckel, 1878

**Order Naviculales Bessey, 1907**

#### ﻿Family Cavinulaceae D.G.Mann, 1990

##### Genus *Cavinula* KD.G.Mann & Stickle, 1844

**144. *Cavinulascutelloides* (W.Smith) Lange-Bertalot, 1996: 31.**

*Naviculascutelloides* W.Smith, 1856: 91.

**Observation.** Main basin: Owino et al. (2020).

Crescent Island Crater: Richardson and Richardson (1972), NC20.

**Occurrence.** Sediment core.

### ﻿Class Bacillariophyceae Haeckel, 1878

**Order Naviculales Bessey, 1907**

#### ﻿Family Diadesmidaceae D.G.Mann, 1990

##### Genus *Diadesmis* Kützing, 1844

**145. *Diadesmisconfervacea* Kützing, 1844: 109, pl. 30 fig. 8.**

*Naviculaconfervacea* (Kützing) Grunow, 1880: pl. 14 fig. 36.

**Observation.** Crescent Island Crater: NC20.

**Occurrence.** Sediment core.

##### Genus *Humidophila* (Lange-Bertalot & Werum) R.L.Lowe, Kociolek, J.R.Johansen, Van de Vijver, Lange-Bertalot & Kopalová, 2014

**146. *Humidophilacontenta* (Grunow) R.L.Lowe, Kociolek, J.R.Johansen, Van de Vijver, Lange-Bertalot & Kopalová, 2014: 357.**

*Naviculacontenta* Grunow, 1885: 109.

*Diadesmiscontenta* (Grunow) D.G.Mann, 1990: 666.

**Observation.** Main basin: Cocquyt and De Wever (2002).

Lake Sonachi: NS93.2-F.

**Occurrence.** Epiphytic, sediment core.

Recent review of this taxon by Van de Vijver et al. (2022) has shown that often valves identified as *H.contenta* belong to a different species, namely *Humidophilasimplex* (E.Reichardt) R.L.Lowe Kociolek, J.R.Johansen, Van de Vijver, Lange-Bertalot & Kopalová (2014: 359). As no photographs or drawings are available of the valves of *H.contenta* that have been sporadically observed in the main basin of Lake Naivasha and in Lake Sonachi, in future studies attention should focus on correct identification of this taxon.

##### Genus *Luticola* D.G.Mann, 1990

**147. *Luticolamutica* (Kützing) D.G.Mann, 1990: 532, 670 figs a–c.**

*Naviculamutica* Kützing 1844: 93, pl. 3 fig. 32.

**Observation.** Crescent Island Crater: Richardson and Richardson (1972), Gasse (1986), Van der Meeren et al. (2019), NC20.

Main basin: Cocquyt and De Wever (2002), Owino et al. (2020), NM91.1-S, NM93.1-S.

Lake Sonachi: NS93.2-F.

**Occurrence.** Plankton, epiphytic, sediment core.

### ﻿Class Bacillariophyceae Haeckel, 1878

**Order Naviculales Bessey, 1907**

#### ﻿Family Amphipleuraceae Grunow, 1990

##### Genus *Frustulia* Rabenhorst, 1853

**148. *Frustuliasaxonica* Rabenhorst, 1853: 50 pl. VII fig. 1.**

Frustuliarhomboidesvar.saxonica (Rabenhorst) De Toni, 1891: 277.

**Observation.** Lake Sonachi: Bachmann (1938), NS93.2-F.

**Occurrence.** Plankton, sediment core.

### ﻿Class Bacillariophyceae Haeckel, 1878

**Order Naviculales Bessey, 1907**

#### ﻿Family Brachysiraceae D.G.Mann, 1990

##### Genus *Brachysira* Kützing, 1836

**149. *Brachysiraexilis* (Kützing) Round & D.G.Mann, 1981: 227.**

**Observation.** Crescent Island Crater: NC20.

**Occurrence.** Sediment core.

**150. Brachysiracf.serians (Brébission) Round & D.G.Mann, 1981: 227.**

*Anomoeoneisserians* (Brébisson) Cleve, 1895: 7.

**Observation.** Lake Sonachi: NS93.2-F.

**Occurrence.** Sediment core.

### ﻿Class Bacillariophyceae Haeckel, 1878

**Order Naviculales Bessey, 1907**

#### ﻿Family Neidiaceae Mereschkowsky, 1903

##### Genus *Neidium* Pfitzer, 1871

**151. *Neidiumaffine* (Ehrenberg) Pfitzer, 1871: 39.**

**Observation.** Crescent Island Crater: NC20.

**Occurrence.** Sediment core.

**152. *Neidiumiridis* (Ehrenberg) Cleve, 1894: 69.**

*Neidiumamphigomphys* (Ehrenberg) Pfitzer, 1871: 39.

Neidiumiridisvar.amphigomphys (Ehrenberg) O’Meara, 1875: 337.

**Observation.** Main basin: Gasse (1986).

Crescent Island Crater: Gasse (1986).

**Occurrence.** Plankton.

**153. *Neidiumproductum* (W.Smith) Cleve, 1894: 69.**

**Observation.** Crescent Island Crater: Gasse 1986.

**Occurrence.** Bottom mud.

**154. *Neidium* sp.**

**Observation.** Crescent Island Crater: Richardson and Richardson (1972).

**Occurrence.** Sediment core.

### ﻿Class Bacillariophyceae Haeckel, 1878

**Order Naviculales Bessey, 1907**

#### ﻿Family Sellaphoraceae Mereschkowsky, 1902

##### Genus *Fallacia* Stickle & D.G.Mann, 1990

**155. *Fallaciapygmaea* (Kützing) Stickle & D.G.Mann, 1990: 668.**

*Naviculapygmaea* Kützing, 1849: 77.

*Lyrellapygmaea* (Kützing) Makarova & Karayeva, 1987: 53, pl. 2 fig. 2.

**Observation.** Main basin: NM91.1-S, NM93.1-S.

**Occurrence.** Sediment core.

### ﻿Class Bacillariophyceae Haeckel, 1878

**Order Naviculales Bessey, 1907**

#### ﻿Family Sellaphoraceae Mereschkowsky, 1902

##### Genus *Sellaphora* Mereschkowsky, 1902

**156. Sellaphoracf.damasii (Hustedt) C.E.Wetzel, Ector, Van de Vijver, Compère & D.G.Mann, 2015: 226.**

*Naviculadamasii* Hustedt, 1962: 203, pl. VII fig. 1322.

Naviculasubcontentavar.africana Hustedt, 1949: 85, pl. 4 figs 27, 28.

**Observation.** Crescent Island Crater: NC20.

**Occurrence.** Sediment core.

**157. *Sellaphoranyassensis* (O.Müller) D.G.Mann, 1989: 2.**

*Naviculanyassensis* O.Müller, 1910: 83, pl. I fig. 5.

Naviculapupulavar.nyassensis Lange-Bertalot, 1985: 89.

**Observation.** Crescent Island Crater: Richardson and Richardson (1972), NC20.

**Occurrence.** Sediment core.

**158. *Sellaphoraperventralis* (Hustedt) A.Tuji, 2003: 71.**

*Naviculaperventralis* Hustedt, 1937: 241, pl. 17 figs 49–50.

**Observation.** Crescent Island Crater: Richardson and Richardson (1972).

**Occurrence.** Sediment core.

**159. Sellaphoracf.pseudoventralis (Hustedt) Chudaev & Gololobova, 2015: 254, figs 17–29.**

*Naviculapseudoventralis* Hustedt, 1953: 631, figs 12, 13.

**Observation.** Crescent Island Crater: NC20.

**Occurrence.** Sediment core.

**160. *Sellaphorapupula* (Kützing) Mereschkovsky, 1902: 187, pl. 4 figs 1–5.**

*Naviculapupula* Kützing, 1844: 93, pl. 30 fig. 40.

**Observation.** Main basin: Gasse (1986), Cocquyt and De Wever (2002), Stoof-Leichsenring et al. (2012), NM91.1-S, NM93.1-S.

Crescent Island Crater: Gasse (1986), Cocquyt and De Wever (2002), NC20.

Lake Oloidien: Gasse (1986).

**Occurrence.** Plankton, bottom mud, sediment core.

**161. *Sellaphorarectangularis* (W.Gregory) Lange-Bertalot & Metzeltin, 1996: 102, pl. 25 figs 10–12, pl. 125 fig. 7.**

Naviculapupulavar.rectangularis (W.Gregory) Cleve & Grunow, 1880: 45.

Naviculapupulaf.rectangularis (W.Gregory) Hustedt, 1961: 121, figs 1254 n–q.

Sellaphorapupulavar.rectangularis (W.Gregory) Mereschkowsky, 1902: 191.

**Observation.** Main basin: NM91.1-S, NM93.1-S.

Crescent Island Crater: Richardson and Richardson (1972), NC20.

**Occurrence.** Sediment core.

**162. Sellaphoracf.seminulum (Grunow) D.G.Mann, 1989: 2.**

*Naviculaseminulum* Grunow, 1860: 552, pl. 2 fig. 3.

**Observation.** Crescent Island Crater: NC20.

**Occurrence.** Sediment core.

**163. *Sellaphora* sp.**

**Observation.** Crescent Island Crater: NC20.

**Occurrence.** Sediment core.

### ﻿Class Bacillariophyceae Haeckel, 1878

**Order Naviculales Bessey, 1907**

#### ﻿Family Pinnulariaceae D.G.Mann, 1990

##### Genus *Caloneis* Cleve, 1894

**164. *Caloneisaequatorialis* Hustedt**, **1921: 148, figs 5, 6.**

**Observation.** Crescent Island Crater: NC20.

**Occurrence.** Sediment core.

**165. *Caloneisbacillum* (Grunow) Cleve, 1894: 99.**

**Observation.** Crescent Island Crater: NC93, NC20.

**Occurrence.** Sediment core.

**166. Caloneiscf.clevei (Lagerstedt) Cleve, 1894: 51.**

**Observation.** Crescent Island Crater: NC93.

**Occurrence.** Sediment core.

**167. *Caloneissilicula* (Ehrenberg) Cleve, 1894: 51.**

**Observation.** Crescent Island Crater: NC93, NC20.

**Occurrence.** Sediment core.

**168. *Caloneisventricosa* (Ehrenberg) F.Meister, 1912: 116, pl. 17 fig. 4.**

**Observation.** Crescent Island Crater: NC20.

**Occurrence.** Sediment core.

##### Genus *Pinnularia* Ehrenberg, 1843

**169. *Pinnulariaacrosphaeria* W.Smith, 1853: 58, pl. XIX fig. 183.**

**Observation.** Main basin: Rich (1932).

**Occurrence.** Bottom mud.

**170. *Pinnulariabiundulata* (O.Müller) Kulikovskiy & Genkal, 2010: 497.**

Pinnulariamicrostauronvar.biundulata O.Müller, 1898: 25.

Pinnulariamicrostauronf.biundulata (O.Müller) Hustedt, 1930: 320, fig. 583.

**Observation.** Main basin: Gasse (1986).

**Occurrence.** Bottom mud.

**171. *Pinnulariaborealis* Ehrenberg, 1843: 420, pl. 4 fig. I.5, pl. 4 fig.V.5.**

**Observation.** Main basin: NM91.1-S, NM93.1-S.

**Occurrence.** Sediment core.

**172. Pinnulariaborealisvar.scalaris (Ehrenberg) Rabenhorst, 1864: 216.**

**Observation.** Main basin: Rich (1932).

**Occurrence.** Bottom mud.

**173. Pinnulariacf.dubitabilis Hustedt, 1949: 105, pl. 6 figs 11–13.**

*Pinnulariaeburnea* Zanon, 1941: 49, pl. III figs 16–18.

**Observation.** Crescent Island Crater: Richardson and Richardson (1972).

**Occurrence.** Sediment core.

**174. *Pinnulariagibba* (Ehrenberg) Ehrenberg, 1843: 315.**

**Observation.** Main basin: Cocquyt and De Wever (2002).

Crescent Island Crater: Gasse (1986).

**Occurrence.** Plankton, epiphytic.

**175. Pinnulariagibbavar.sancta (Grunow ex Cleve) F.Meister, 1932: 43, fig. 140.**

**Observation.** Main basin: Stoof-Leichsenring et al. (2012), NM93.1-S, and cf. this taxon in NM91.1-S.

**Occurrence.** Plankton, epiphytic.

**176. *Pinnulariainterrupta* W.Smith, 1853: 59, pl. 19 fig. 184.**

*Naviculainterrupta* (W.Smith) Grunow, 1860: 521.

**Observation.** Main basin: Rich (1932).

**Occurrence.** Bottom mud.

**177. *Pinnulariainterruptiformis* Krammer, 2000: 109, pl. 85 figs 1–9.**

Pinnulariabicepsf.petersenii R.Ross, 1947: 201, pl. 9 fig. 11.

**Observation.** Crescent Island Crater: NC20.

Lake Oloidien: Gasse (1986).

**Occurrence.** Bottom mud, sediment core.

**178. *Pinnulariamesolepta* (Ehrenberg) W.Smith, 1853: 58, pl. 19 fig. 182.**

*Naviculamesolepta* Ehrenberg, 1843: 419, pl. 4/2 fig. 4.

**Observation.** Main basin: Rich (1932).

**Occurrence.** Bottom mud.

**179. *Pinnulariamajor* (Kützing) Rabenhorst, 1853: 42, pl. 6 fig. 5.**

**Observation.** Main basin: Owino et al. (2020).

Crescent Island Crater: Gasse (1986).

Lake Oloidien: Gasse (1986).

**Occurrence.** Plankton, bottom mud, sediment core.

**180. *Pinnulariasubcapitata* W.Gregory, 1856: 9, pl. 1 fig. 30.**

**Observation.** Crescent Island Crater: Gasse (1986).

**Occurrence.** Plankton.

**181. *Pinnulariaviridis* (Nitzsch) Ehrenberg, 1843: 305, 315, 385, pl. 1 fig. 7.**

**Observation.** Main basin: Owino et al. (2020).

**Occurrence.** Sediment core.

**Remark.** Unidentified *Pinnularia* taxa are reported from Crescent Island (core NC20) and from the main basin (Cocquyt and De Wever 2002 and cores NM91.1-S and NM93.1-S.).

### ﻿Class Bacillariophyceae Haeckel, 1878

**Order Naviculales Bessey, 1907**

#### ﻿Family Diploneidaceae D.G.Mann, 1990

##### Genus *Diploneis* Ehrenberg ex Cleve, 1894

**182. *Diploneiselliptica* (Kützing) Cleve, 1894: 92.**

**Observation.** Crescent Island Crater: NC20.

**Occurrence.** Sediment core.

**183. *Diploneisovalis* (Hilse) Cleve, 1891: 44, pl. 2 fig. 13.**

**Observation.** Crescent Island Crater: Richardson and Richardson (1972).

**Occurrence.** Sediment core.

**184. *Diploneispseudovalis* Hustedt, 1930: 253, fig. 403.**

**Observation.** Main basin: Stoof-Leichsenring et al. (2012).

**Occurrence.** Sediment core.

**185. *Diploneis* sp.**

**Observation.** Crescent Island Crater: Richardson and Richardson (1972).

**Occurrence.** Sediment core.

An unidentified *Diploneis* taxon was also observed in the core taken from Crescent Island Crater (core NC20) and from the main basin (core NM91.1-S.).

### ﻿Class Bacillariophyceae Haeckel, 1878

**Order Naviculales Bessey, 1907**

#### ﻿Family Naviculaceae Kütizing, 1844

##### Genus *Adlafia* Gerd Moser, Lange-Bertalot & Metzeltin, 1998

**186. *Adlafiapseudomuralis* (Hustedt) J.Y.Li & Y.Z.Qi, nom. inval., 2018: 5, pl. 1 fig. 6.**

*Naviculapseudomuralis* Hustedt, 1937: 245, pl. 19 figs 25–27.

*Fallaciapseudomuralis* (Hustedt) D.G.Mann, 1990: 669.

**Observation.** Crescent Island Crater: Gasse (1986).

**Occurrence.** Plankton.

##### Genus *Hippodonta* Lange-Bertalot, Witkowski & Metzeltin, 1996

**187. Hippodontacf.costulata (Grunow) Lange-Bertalot, Metzeltin & Witkowski, 1996: 254, pl. 1 figs 6, 7, pl. 3 fig. 5, pl. 4 figs 6–9.**

*Naviculacostulata* Grunow, 1880: 27.

**Observation.** Crescent Island Crater: NC20.

**Occurrence.** Sediment core.

##### Genus *Mayamaea* Lange-Bertalot, 1997

**188. *Mayamaeapermitis* (Hustedt) Bruder & Medlin, 2008: 327.**

*Naviculapermitis* Hustedt, 1945: 919, pl. 41 figs 8, 9.

**Observation.** Main basin: Gasse (1896).

**Occurrence.** Bottom mud.

##### Genus *Navicula* Bory, 1822

**189. *Naviculabarbarica* Hustedt, 1949: 97, pl. IV figs 14–17.**

**Observation.** Crescent Island Crater: Richardson and Richardson (1972).

**Occurrence.** Sediment core.

**190. *Naviculacapitatoradiata* H.Germain ex Gasse, 1986: 86, pl. 19 figs 8–9.**

**Observation.** Crescent Island Crater: core NC20.

**Occurrence.** Sediment core.

**191. Naviculacarivar.cincta (Ehrenberg) Lange-Bertalot, 1980: 37, pl. 7 figs 1–23.**

**Observation.** Main basin: Gasse (1986).

Crescent Island Crater: Gasse (1986).

Lake Oloidien: Gasse (1986).

**Occurrence.** Plankton, bottom mud.

**192. *Naviculacryptocephala* Kützing, 1844: 95, pl. 3 figs 20, 26.**

**Observation.** Main basin: Gasse ((1986), Cocquyt and De Wever (2002), Stoof-Leichsenring et al. (2011), Stoof-Leichsenring et al. (2012).

Crescent Island Crater: Richardson and Richardson (1972), Gasse (1986).

**Occurrence.** Plankton, epiphytic, bottom mud, sediment core.

**193. *Naviculacryptotenella* Lange-Bertalot, 1985: 62, pl. 18 figs 22, 23, pl. 19 figs 1–10, pl. 27 fig. 1.**

**Observation.** Main basin: NM91.1-S, NM93.1-S.

Crescent Island Crater: NC20.

**Occurrence.** Sediment core.

**194. Naviculacf.exigua W.Gregory nom. illeg., 1854: 99.**

**Observation.** Main basin: NM91.1-S, NM93.1-S.

Crescent Island Crater: NC20.

**Occurrence.** Sediment core.

According to Guiry and Guiry (2022) the taxonomic and nomenclatural status of *Naviculaexigua* is in some way unresolved and requires further investigation.

**195. Naviculacf.glomus J.R.Carter, 1981: 578, pl. 13 fig. 17.**

**Observation.** Crescent Island Crater: NC20.

**Occurrence.** Sediment core.

**196. *Naviculaminima* Grunow, 1880: pl. XIV fig. 15.**

**Observation.** Crescent Island Crater: Richardson and Richardson (1972).

**Occurrence.** Sediment core.

**197. *Navicularadiosa* Kützing, 1844: 91, pl. 4 fig. 23.**

Navicularadiosavar.acuta (W.Smith) Grunow, 1860: 526.

**Observation.** Main basin: Rich (1932), Gasse (1986), Stoof-Leichsenring et al. (2011), Stoof-Leichsenring et al. (2012).

Crescent Island Crater: Richardson and Richardson (1972), NC20.

**Occurrence.** Bottom mud, sediment core.

**198. Navicularadiosaf.divergentissima Manguin, 1952: 26, fig. 54.**

*Navicularadiosa* [var. acuta] f. divergentissima Manguin, 1952: 26, fig. 54.

**Observation.** Crescent Island Crater: NC20.

**Occurrence.** Sediment core.

According to Guiry and Guiry (2022) the taxonomic status of this taxon requires further investigation.

**199. *Navicularadiosafallax* Lange-Bertalot, 1993: 131, pl. 52 figs 1–3.**

Navicularadiosavar.parva J.H.Wallace, 1960: 3, pl. 1 fig. 5.

**Observation.** Main basin: NM93.1-S, and cf. this taxon in NM91.1-S.

**Occurrence.** Sediment core.

**200. Naviculaseminuloidesvar.sumatrensis Hustedt, 1937: 239, pl. 17 figs 32, 33.**

**Observation.** Crescent Island Crater: Richardson and Richardson (1972).

**Occurrence.** Sediment core.

Richardson and Richardson (1972) mentioned the name of this taxon as Naviculaseminuloidesvar.sumatrana.

**201. *Navicularhynchocephala* Kützing, 1844: 152, pl. 30 fig. 35.**

**Observation.** Crescent Island Crater: NC20.

**Occurrence.** Sediment core.

**202. *Navicularostellata* Kützing, 1844: 95, pl. 3 fig. 65.**

Navicularhynchocephalavar.rostellata (Kützing) Cleve & Grunow, 1880: 33.

Naviculaviridulavar.rostellata (Kützing) Cleve, 1895: 15.

**Observation.** Crescent Island Crater: NC20.

**Occurrence.** Sediment core.

**203. Naviculaaff.tenella Brébisson ex Kützing, 1849: 74.**

Navicularadiosavar.tenella (Brébisson ex Kützing) Van Heurck, 1885: 84.

**Observation.** Main basin: NM93.1-S.

Crescent Island Crater: Gasse (1986).

**Occurrence.** Plankton, sediment core.

**204. *Naviculatrivialis* Lange-Bertalot, 1980: 31, pl. 1 figs 5–9.**

**Observation.** Main basin: NM91.1-S.

Crescent Island Crater: Richardson and Richardson (1972).

**Occurrence.** Sediment core.

**205. *Naviculaveneta* Kützing, 1844: 95, pl. 30 fig. 76.**

Naviculacryptocephalavar.veneta (Kützing) Rabenhorst, 1864: 198.

Naviculacryptocephalaf.veneta (Kützing) Hustedt, 1957: 290.

**Observation.** Main basin: Cocquyt and De Wever (2002).

Crescent Island Crater: Gasse (1986).

**Occurrence.** Plankton, epiphytic.

**206. *Naviculaviridula* (Kützing) Ehrenberg, 1836: 53.**

**Observation.** Main basin: NM91.1-S, NM93.1-S.

Crescent Island Crater: Richardson and Richardson (1972).

**Occurrence.** Sediment core.

**207. *Naviculazanonii* Hustedt, 1949: 792, pl. 5 figs 1–5.**

**Observation.** Main basin: Gasse (1986), NM91.1-S.

**Occurrence.** Bottom mud.

**Remark.**Owino et al. (2020) reported, besides an unidentified *Navicula*, also “*Naviculagranatum*”. Probably a typing error occurred as a *Navicula* with the epithet *granatum* does not exit. In core NM91.1-S, a small *Navicula* was observed. However, we do not know to which genus split off from *Navicula* s.l. this taxon currently belongs.

##### Genus *Navigiolum* Lange-Bertalot, Cavacini, Tagliaventi & Alfinito, 2003

**208. *Navigiolumadamantiforme* (R.E.M.Archibald) J.C.Taylor & Lange-Bertalot, 2006: 177, figs 2a–k.**

*Naviculaadamantiforme* R.E.M.Archibald, 1966: 256, figs 5, 6.

**Observation.** Crescent Island Crater: core NC20.

**Occurrence.** Sediment core.

### ﻿Class Bacillariophyceae Haeckel, 1878

**Order Naviculales Bessey, 1907**

#### ﻿Family Stauroneidaceae D.G.Mann, 1990

##### Genus *Craticula* Grunow, 1868

**209. *Craticulabuderi* (Hustedt) Lange-Bertalot, 2000: 101, pl. 58 fig. 3.**

*Naviculabuderi* Hustedt, 1954: 279, figs 11–15.

**Observation.** Lake Sonachi: NS93.2-F.

**Occurrence.** Sediment core.

**210. *Craticulaambigua* (Ehrenberg) D.G.Mann, 1990: 666.**

*Naviculaambigua* Ehrenberg, 1843: 417, pl. 2/2 fig. 9.

Naviculacuspidatavar.ambigua (Ehrenberg) Kirchner, 1878: 178.

**Observation.** Main basin: Bachmann (1938).

Crescent Island Crater: NC93, NC20.

**Occurrence.** Littoral, sediment core.

**211. *Craticulacuspidata* (Kützing) D.G.Mann, 1990: 666.**

*Naviculacuspidata* (Kützing) Kützing, 1844: 94, pl. 3 figs 34, 37.

**Observation.** Main basin: Gasse (1986).

Lake Sonachi: NS93.2-F.

**Occurrence.** Plankton, sediment core.

**212. Craticulacuspidatavar.major (F.Meister) Czarnecki, 1994: 96.**

Naviculacuspidatavar.major F.Meister, 1912: 134, pl. 20 fig. 10.

**Observation.** Main basin: Rich (1932).

**Occurrence.** Bottom mud.

**213. *Craticulaelkab* (O.Müller ex O.Müller) Lange-Bertalot, Kusber & Cocquyt, 2007: 119.**

*Naviculaelkab* O.Müller ex O.Müller, 1910: 76 figs. 19–22.

**Observation.** Main basin: Cocquyt and De Wever (2002), Stoof-Leichsenring et al. (2011), Stoof-Leichsenring et al. (2012), NM91.1-S, NM93.1-S.

Crescent Island Crater: Richardson and Richardson (1972), Gasse (1986), Van der Meeren et al. (2019), NC20.

Lake Oloidien: Gasse (1986), Verschuren et al. (1999b), Verschuren et al. (2000b).

Lake Sonachi: Gasse (1986), Cocquyt and De Wever (2002), NS93.2-F.

**Occurrence.** Epiphytic, bottom mud, sediment core.

**214. *Craticulahalophila* (Grunow) D.G.Mann, 1990: 666.**

Naviculacuspidatavar.halophila Grunow, 1885: 100, suppl. pl. B fig. 30.

*Naviculahalophila* (Grunow) Cleve, 1894: 109.

Naviculahalophilaf.robusta Hustedt, 1959: 401, figs. 1–3.

**Observation.** Main basin: Gasse (1986), Stoof-Leichsenring et al. (2012), NM91.1-S, NM93.1-S.

Lake Sonachi: Gasse (1986), NS93.2-F.

**Occurrence.** Plankton, bottom mud, sediment core.

**215. Craticulaaff.minusculoides (Hustedt) Lange-Bertalot, 2001: 115.**

*Naviculaminusculoides* Hustedt, 1942: 88, fig. 5.

**Observation.** Crescent Island Crater: Gasse (1986).

**Occurrence.** Bottom mud.

**216. *Craticulamolestiformis* (Hustedt) Mayama, 1999: 2.**

*Naviculamolestiformis* Hustedt, 1949: 86, pl. 5 fig. 9.

*Naviculatwymaniana* R.E.M.Archibald, 1966: 264, figs 41–43.

**Observation.** Crescent Island Crater: Gasse (1986).

**Occurrence.** Plankton.

**217. *Craticulaperrotettii* Grunow, 1868: 20, pl. 1 fig. 1.**

*Naviculaperrotettii* (Grunow) Cleve, 1894: 110, pl. 3 fig. 12.

**Observation.** Crescent Island Crater: NC20.

**Occurrence.** Sediment core.

**Remark.**Rich (1932) reported Naviculacuspidatavar.major F.Meister 1912: 134, pl. 20 fig. 10. (Craticulacuspidatavar.major (Meister) Czarnecki 1994: 96) in bottom mud from the main basin of Lake Naivasha. According the Guiry and Guiry (2022) the taxonomic status of this taxon requires further investigation, but the valves observed by Rich (1932) are very probably one of the larger *Craticula* species observed in Lake Naivasha, e.g., *Craticulacuspidata* or *Craticulaperrotettii*.

##### Genus *Dorofeyukea* Kulikovskiy, Maltsev, S.A.Andreeva, T.Ludwig & Kociolek, 2019

**218. *Dorofeyukeakotschyi* (Grunow) Kulikovskiy, Kociolek, Tusset & T.Ludwig, 2019: 178, figs 5–7.**

*Naviculakotschyi* Grunow, 1860: 538, pl. 2 fig. 12.

*Luticolakotschyi* (Grunow) J.C.Taylor, W.C.Harding & C.G.M.Archibald, nom. inval., 2007: 50.

**Observation.** Crescent Island Crater: NC20.

**Occurrence.** Sediment core.

##### Genus *Fistulifera* Lange-Bertalot, 1997

**219. *Fistuliferapelliculosa* (Kützing) Lange-Bertalot, 1997: 73, figs 28–31.**

*Naviculapelliculosa* (Kützing) Hilse, 1863: 68.

**Observation.** Crescent Island Crater: Gasse (1986).

**Occurrence.** Plankton.

##### Genus *Stauroneis* Ehrenberg, 1843

**220. *Stauroneisphoenicenteron* (Nitzsch) Ehrenberg, 1843: 311, pl. 2 fig. 1, pl. 3 fig. 3.**

Stauroneisphoenicenteronvar.genuina Cleve 1894: 149.

**Observation.** Main basin: Rich (1932).

**Occurrence.** Bottom mud.

### ﻿Class Bacillariophyceae Haeckel, 1878

**Order Thalassiophysales D.G.Mann, 1990**

#### ﻿Family Catenulaceae Mereschkowsky, 1902

##### Genus *Amphora* Ehrenberg ex Kützing, 1844

**221. *Amphoracopulata* (Kützing) Schoeman & R.E.M.Archibald, 1986: 429, figs 1–13, 30–34.**

Amphoraovalisvar.libyca (Ehrenberg) Cleve, 1895: 104 pro parte.

*Amphoralibyca* Ehrenberg, 1841: 205 pro parte.

**Observation.** Main basin: Rich (1932), Cocquyt and De Wever (2002), Stoof-Leichsenring et al. (2011), Stoof-Leichsenring et al. (2012), NM91.1-S, NM93.1-S.

Crescent Island Crater: Richardson and Richardson (1972), NC20.

**Occurrence.** Bottom mud, sediment core

Tropical African specimens of *Amphoralibyca* (Ehrenberg) Cleve, 1895: 104 (synonym: Amphoraovalisvar.libyca (Ehrenberg) Cleve, 1895: 104) were often put in synonymy with *Amphoracopulata*. However, further investigation of the valves observed in Lake Naivasha and its satellite lakes is needed to see if these valves belongs to a cluster of different species. The revision work of the genus *Amphora* by Levkov (2009) suggests that we may indeed be dealing here with several species, as was the case for Lake Tanganyika where many new species were described.

**222. *Amphoragouwsii* Cholnoky, 1953: 352, fig. 1.**

**Observation.** Crescent Island Crater: Richardson and Richardson (1972).

**Occurrence.** Sediment core.

According to Guiry and Guiry (2022) the taxonomic status of this taxon requires further investigation.

**223. *Amphoraovalis* (Kützing) Kützing, 1844: 107, pl. 5 figs 35, 39.**

**Observation.** Crescent Island Crater: Richardson and Richardson (1972), NC20.

**Occurrence.** Sediment core.

**224. *Amphorapediculus* (Kützing) Grunow, 1875: pl. 26 fig. 99.**

Amphoraovalisvar.pediculus (Kützing) Van Heurck, 1885: 59.

**Observation.** Main basin: Gasse (1986).

Crescent Island Crater: Richardson and Richardson (1972).

Lake Sonachi: Gasse (1986).

**Occurrence.** Plankton, bottom mud, sediment core.

**Remark.**Owino et al. (2020) reported a *Amphora* sp., probably one of the above mentioned taxa or one of the following *Halamphora* taxa, in the sediment core of the Main basin they studied.

##### Genus *Halamphora* (Cleve) Mereschkowsky, 1903

**225. *Halamphorathermalis* (Hustedt) Levkov, 2009: 235, pl. 108 figs 19–39, pl. 230 figs 1–6.**

*Amphorathermalis* Hustedt, 1949: 111, pl. 11 figs 1–3.

*Amphorahartii* Cholnoky, 1963: 30, figs 1–3.

**Observation.** Lake Sonachi: NS93.2-F.

**Occurrence.** Sediment core.

**226. *Halamphorathumensis* (Ant.Mayer) Levkov, 2009: 236, pl. 98 figs 10–20, pl. 219 fig. 6.**

Amphoracoffeiformisvar.thumensis Ant.Mayer, 1919: 208, pl. 9 fig. 69.

*Cymbellathumensis* (Ant.Mayer) Hustedt, 1945: 938.

**Observation.** Crescent Island Crater: Richardson and Richardson (1972).

**Occurrence.** Sediment core.

**227. *Halamphoraveneta* (Kützing) Levkov, 2009: 242, pl. 94 figs 9–19, pl. 102 figs 17–30, pl. 217 figs 1–5, pl. 218 figs 1–5.**

*Amphoraveneta* Kützing, 1844: 108, pl. 3 fig. 25.

**Observation.** Main basin: Gasse (1986), Cocquyt and De Wever (2002), NM91.1-S, NM93.1-S.

Crescent Island Crater: Richardson and Richardson (1972), Gasse (1986).

Lake Oloidien: Gasse (1986).

Lake Sonachi: Gasse (1986).

**Occurrence.** Epiphytic, bottom mud, sediment core.

### ﻿Class Bacillariophyceae Haeckel, 1878

**Order Bacillariales Hendey, 1937**

#### ﻿Family Bacillariaceae Ehrenberg, 1831

##### Genus *Hantzschia* Grunow, 1877

**228. *Hantzschiaamphioxys* (Ehrenberg) Grunow, 1880: 103.**

**Observation.** Main basin: Gasse (1986), Cocquyt and De Wever (2002), NM91.1-S, NM93.1-S.

Crescent Island Crater: Richardson and Richardson (1972), Gasse 1986, NC20.

Lake Oloidien: Gasse (1986).

Lake Sonachi: NS93.2-F.

**Occurrence.** Epiphytic, bottom mud, sediment core.

**229. Hantzschiaamphioxysvar.africana Hustedt, 1921: 131, 197, pl. 1 fig. 25.**

**Observation.** Main basin: Gasse (1986), Cocquyt and De Wever (2002), NM93.1-S.

Crescent Island Crater: Richardson and Richardson (1972), Gasse (1986), NC20.

Lake Oloidien: Gasse (1986).

Lake Sonachi: NS93.2-F.

**Occurrence.** Epiphytic, bottom mud, sediment core.

##### Genus *Nitzschia* Hassall, 1845

**230. *Nitzschiaaccommodata* Hustedt, 1949: 139, pl. 12 figs 27–31, 34, 35.**

**Observation.** Crescent Island Crater: Richardson and Richardson (1972).

**Occurrence.** Sediment core.

**231. *Nitzschiaacicularis* (Kützing) W.Smith, 1853: 43, pl. 15 fig. 122.**

**Observation.** Crescent Island Crater: NC20.

**Occurrence.** Sediment core.

**232. Nitzschiacf.adapta Hustedt, 1949: 135, pl. XII figs 3–6.**

**Observation.** Crescent Island Crater: Richardson and Richardson (1972), NC20.

**Occurrence.** Sediment core.

**233. *Nitzschiaamphibia* Grunow, 1862: 574, pl. 28 fig. 23.**

**Observation.** Main basin: Gasse (1986), Cocquyt and De Wever (2002), NM91.1-S.

Crescent Island Crater: Richardson and Richardson (1972), Gasse (1986), NC20.

Lake Oloidien: Gasse (1986).

**Occurrence.** Plankton, bottom mud, sediment core.

**234. Nitzschiaamphibiaf.frauenfeldii (Grunow) Lange-Bertalot, 1987: 5, pl. 37 figs 15–22.**

**Observation.** Crescent Island Crater: NC20.

**Occurrence.** Sediment core.

According to Guiry and Guiry (2022) the taxonomic or nomenclatural status (or both) of this entity is in some way unresolved and requires further investigation.

**235. Nitzschiacf.archibaldii Lange-Bertalot, 1980: 44, pl. 1 figs 14–18, pl. 7 figs 115–121.**

**Observation.** Main basin: Gasse (1986).

Crescent Island Crater: Gasse (1986).

Lake Oloidien: Gasse (1986).

Lake Sonachi: NS93.2-F.

**Occurrence.** Plankton, bottom mud, sediment core.

**236. *Nitzschiabacata* Hustedt, 1937: 485, pl. 41 figs 30–33.**

**Observation.** Crescent Island Crater: Richardson and Richardson (1972), NC20.

**Occurrence.** Sediment core.

**237. *Nitzschiabrevissima* Grunow, 1880: 485, pl. LXVII fig. 4.**

Nitzschiaobtusavar.brevissima Grunow, 1885: 180.

**Observation.** Crescent Island Crater: Richardson and Richardson (1972).

**Occurrence.** Sediment core.

**238. *Nitzschiaclausii* Hantzsch, 1860: 40, pl. 6 fig. 7.**

**Observation.** Lake Sonachi: NS93.2-F.

**Occurrence.** Sediment core.

**239. *Nitzschiacommunis* Rabenhorst, 1860: no. 949.**

**Observation.** Main basin: NM93.1-S, and cf. this taxon in NM91.1-S.

Lake Sonachi: NS93.2-F.

**Occurrence.** Sediment core.

**240. *Nitzschiaconfinis* Hustedt, 1949: 145, pl. 11 figs 49–54, pl. 13 figs 84–90.**

**Observation.** Crescent Island Crater: Richardson and Richardson (1972).

**Occurrence.** Sediment core.

**241. *Nitzschiadissipata*** (**Kützing) Rabenhorst, 1860: no. 968.**

**Observation.** Main basin: Owino et al. (2020).

Crescent Island Crater: NC20.

**Occurrence.** Sediment core.

**242. *Nitzschiafiliformis* (W.Smith) Van Heurck, 1896: 406, pl. 33 fig. 882.**

**Observation.** Crescent Island Crater: NC20.

**Occurrence.** Sediment core.

**243. *Nitzschiafonticola*** (**Grunow) Grunow, 1881: pl. LXIX figs 15–20.**

**Observation.** Crescent Island Crater: Richardson and Richardson (1972), NC20.

Lake Sonachi: NS93.2-F.

**Occurrence.** Sediment core.

**244. Nitzschiafonticolavar.pelagica Hustedt, in A.W.F.Schmidt 1924: pl 349 figs 15–16.**

**Observation.** Crescent Island Crater: NC20.

**Occurrence.** Sediment core.

**245. *Nitzschiafrustulum* (Kützing) Grunow, 1880: 98.**

**Observation.** Main basin: Stoof-Leichsenring et al. (2011), Stoof-Leichsenring et al. (2012).

Crescent Island Crater: Richardson and Richardson (1972), NC20.

Lake Sonachi: NS93.2-F.

**Occurrence.** Sediment core.

**246. Nitzschiaaff.frustulum (Kützing) Grunow, 1880: 98.**

**Observation.** Main basin: Cocquyt and De Wever (2002).

Crescent Island Crater: Gasse (1986), Cocquyt and De Wever (2002).

**Occurrence.** Epiphytic, bottom mud.

**247. Nitzschiafrustulumvar.perpusilla (Rabenhorst) Van Heurck, 1885: 184.**

**Observation.** Crescent Island Crater: Richardson and Richardson (1972).

**Occurrence.** Sediment core.

**248. *Nitzschiagoetzeana* O.Müller, 1905: 176, pl. II fig. 20.**

**Observation.** Crescent Island Crater: Richardson and Richardson (1972).

**Occurrence.** Sediment core.

**249. *Nitzschiagracilis* Hantzsch, 1860: 40, pl. 6 fig. 8.**

**Observation.** Main basin: Gasse (1986), NM91.1-S, NM93.1-S.

Crescent Island Crater: Gasse (1986).

Lake Oloidien: Gasse (1986).

**Occurrence.** Plankton, bottom mud, sediment core.

**250. Nitzschiacf.inconspicua Grunow, 1862: 579, pl. 28/12 fig. 25.**

**Observation.** Crescent Island Crater: NC20.

Lake Sonachi: NS93.2-F.

**Occurrence.** Sediment core.

**251. Nitzschiacf.intermedia Hantzsch ex Cleve & Grunow, 1880: 95.**

**Observation.** Main basin: Gasse (1986), Stoof-Leichsenring et al. (2011), Stoof-Leichsenring et al. (2012), NM91.1-S, NM93.1-S.

Crescent Island Crater: Gasse (1986), NC20.

Lake Oloidien: Gasse (1986).

Lake Sonachi: NS93.2-F.

**Occurrence.** Plankton, bottom mud, sediment core.

**252. *Nitzschialacustris* Hustedt, 1922: 166.**

**Observation.** Crescent Island Crater: Richardson and Richardson (1972).

Main basin: Owino et al. (2020).

Lake Oloidien: Gasse (1986).

**Occurrence.** Bottom mud, sediment core.

**253. *Nitzschialancettula* O.Müller, 1905: 175, pl. 2 fig. 15.**

**Observation.** Main basin: Cocquyt and De Wever (2002).

Crescent Island Crater: Richardson and Richardson (1972), Gasse (1986), NC20.

**Occurrence.** Bottom mud, epiphytic, sediment core.

**254. *Nitzschialatens* Hustedt, 1949: 148, pl. XII figs 30, 31.**

**Observation.** Main basin: Cocquyt and De Wever (2002), NM93.1-S, and cf. this taxon in NM93.1-S.

Crescent Island Crater: NC20.

Lake Sonachi: NS93.2-F.

**Occurrence.** Epiphytic, sediment core.

**255. *Nitzschialinearis* W.Smith, 1853: 39, pl. XIII fig. 110.**

**Observation.** Main basin: Bachmann (1938), Stoof-Leichsenring et al. (2011), Stoof-Leichsenring et al. (2012), NM93.1-S, and cf. this taxon in NM91.1-S.

Crescent Island Crater: NC20.

Lake Oloidien: Gasse (1986).

**Occurrence.** Plankton, bottom mud, sediment core.

**256. Nitzschiacf.mediocris Hustedt, 1949: 149, pl. XIII figs 21–24.**

**Observation.** Crescent Island Crater: Richardson and Richardson (1972), NC20.

**Occurrence.** Sediment core.

**257. *Nitzschiaobtusa* W.Smith, 1853: 39, pl. XIII fig. 109.**

**Observation.** Lake Sonachi: NS93.2-F.

**Occurrence.** Sediment core.

**258. *Nitzschiapalea* (Kützing) W.Smith, 1856: 89.**

**Observation.** Main basin: Gasse (1986), Stoof-Leichsenring et al. (2011), Stoof-Leichsenring et al. (2012), Owino et al. (2020), NM91.1-S, NM93.1-S.

Crescent Island Crater: Richardson and Richardson (1972), Gasse (1986), Van der Meeren et al. (2019), NC20.

Lake Oloidien: Gasse (1986).

Lake Sonachi: Verschuren et al. (1999a), Verschuren et al. (2000b), NS93.2-F.

**Occurrence.** Plankton, bottom mud, sediment core.

**259. Nitzschiapaleavar.debilis (Kützing) Grunow, 1880: 96.**

**Observation.** Main basin: Gasse (1986), NM91.1-S, NM93.1-S.

Crescent Island Crater: Gasse (1986), Van der Meeren et al. (2019), NC20.

Lake Oloidien: Gasse (1986).

Lake Sonachi: NS93.2-F.

**Occurrence.** Plankton, bottom mud, sediment core.

**260. Nitzschiapaleavar.tenuirostris Grunow, 1881: pl. 69 fig. 13.**

**Observation.** Main basin: NM91.1-S, NM93.1-S.

Crescent Island Crater: NC20.

**Occurrence.** Sediment core.

**261. Nitzschiapaleavar.tropica Hustedt nom. illeg., 1949: 147 pl, XIII figs 26–29.**

**Observation.** Crescent Island Crater: NC20.

**Occurrence.** Sediment core.

This taxon is different from Nitzschiapaleavar.tropica Grunow (1880: 96) and needs further investigation.

**262. *Nitzschiapaleacea* (Grunow) Grunow, 1881: pl. LXVIII figs 9, 10.**

**Observation.** Main basin: NM91.1-S, NM93.1-S.

Lake Sonachi: NS93.2-F.

**Occurrence.** Sediment core.

**263. *Nitzschiaperminuta* Grunow, 1881: pl. LXIX figs 4, 7.**

Nitzschiafrustulumvar.perminuta Grunow, 1881: pl. LXVIII fig. 31.

**Observation.** Crescent Island Crater: Richardson and Richardson (1972).

**Occurrence.** Sediment core.

**264. Nitzschiacf.pura Hustedt, 1954: 480, figs 70–75.**

**Observation.** Main basin: NM91.1-S, NM93.1-S.

Crescent Island Crater: NC20.

Lake Sonachi: NS93.2-F.

**Occurrence.** Sediment core.

**265. Nitzschiacf.pusilla Grunow, 1862: 579, pl. 28 fig. 11.**

**Observation.** Crescent Island Crater: Van der Meeren et al. 2019).

**Occurrence.** Sediment core.

**266. *Nitzschiarecta* Hantzsch ex Rabenhorst, 1862: no. 1283.**

**Observation.** Main basin: Owino et al.(2020), NM91.1-S, NM93.1-S.

Crescent Island Crater: NC20.

**Occurrence.** Sediment core.

**267. *Nitzschiarostellata* Hustedt, 1956: 127, figs. 69, 70.**

**Observation.** Main basin: NM91.1-S, NM93.1-S.

**Occurrence.** Sediment core.

**268. *Nitzschiasigma* (Kützing) W.Smith, 1853: 39, pl. XIII fig. 108.**

**Observation.** Main basin: NM93.1-S, and cf. this taxon in NM91.1-S.

Crescent Island Crater: NC20.

Lake Sonachi: NS93.2-F.

**Occurrence.** Sediment core.

**269. *Nitzschiasigmoidea* (Nitzsch) W.Smith, 1853: 38, pl. XIII fig. 104.**

**Observation.** Lake Sonachi: Bachmann (1938).

**Occurrence.** Plankton.

**270. *Nitzschiaspiculoides* Hustedt, 1949: 151, pl. 13 figs 5, 6.**

**Observation.** Crescent Island Crater: NC20.

**Occurrence.** Sediment core.

Richardson and Richardson (1972) reported Nitzschiacf.spiculoides from a sediment core taken in Crescent Island.

**271. *Nitzschiasubacicularis* Hustedt, 1938: 490, pl. 41 fig. 12.**

**Observation.** Main basin: Stoof-Leichsenring et al. (2011), Stoof-Leichsenring et al. (2012), Owino et al. (2020), NM91.1-S, NM93.1-S.

Crescent Island Crater: NC20.

Lake Sonachi: NS93.2-F.

**Occurrence.** Sediment core.

Richardson and Richardson (1972) reported Nitzschiacf.subacicularis from a sediment core taken in Crescent Island Crater.

**272. *Nitzschiasubcommunis* Hustedt, 1949: 146, pl. XI 41 figs 55–58, pl. XIII figs 101–106.**

**Observation.** Crescent Island Crater: NC20.

**Occurrence.** Sediment core.

Richardson and Richardson (1972) reported Nitzschiacf.subcommunis from a sediment core taken in Crescent Island Crater.

**273. *Nitzschiasubrostrata* Hustedt, 1942: 137, figs 313–319.**

**Observation.** Main basin: Gasse (1986), NM91.1-S, NM93.1-S.

Crescent Island Crater: Richardson and Richardson (1972), NC20.

**Occurrence.** Bottom mud, sediment core.

**274. Nitzschiacf.subrostrata Hustedt, 1942: 137, figs 313–319.**

**Observation.** Crescent Island Crater: Richardson and Richardson (1972).

**Occurrence.** Sediment core.

**275. *Nitzschiatarda* Hustedt, 1949: 138, pl. XII figs 24, 25.**

**Observation.** Crescent Island Crater: Richardson and Richardson (1972), NC20.

**Occurrence.** Sediment core.

**276. *Nitzschiathermalis* (Ehrenberg) Auerswald, 1861: no. 1064a.**

**Observation.** Main basin: Rich (1932), Gasse (1986).

Crescent Island Crater: NC20.

Lake Oloidien: Gasse (1986).

**Occurrence.** Plankton, bottom mud, sediment core.

**277. Nitzschiacf.tropica Hustedt, 1949: 147, pl. XI figs 34–48.**

**Observation.** Crescent Island Crater: Richardson and Richardson (1972).

**Occurrence.** Sediment core.

**278. *Nitzschiavanoyei* Cholnoky, 1954: 420, figs 75–81.**

**Observation.** Crescent Island Crater: NC20.

**Occurrence.** Sediment core.

Richardson and Richardson (1972) reported Nitzschiacf.vanoyei from a sediment core taken in Crescent Island Crater.

**279. *Nitzschiavitrea* G.Norman, 1861: 7, pl. 2 fig. 4.**

**Observation.** Main basin: Cocquyt and De Wever (2002), NM91.1-S, NM93.1-S.

**Occurrence.** Epiphytic.

##### Genus *Tryblionella* W.Smith, 1853

**280. *Tryblionellacalida* (Grunow) D.G.Mann, 1990: 678.**

*Nitzschiacalida* Grunow, 1880: 75.

Nitzschiatryblionellavar.calida (Grunow) Van Heurck, 1885: 171.

**Observation.** Crescent Island Crater: NC20.

**Occurrence.** Sediment core.

**281. *Tryblionellaumbilicata* (Hustedt) D.G.Mann, 1990: 679.**

*Nitzschiaumbilicata* Hustedt, 1949: 129, pl. XI fig. 65.

**Observation.** Main basin: NM91.1-S, NM93.1-S.

**Occurrence.** Sediment core.

### ﻿Class Bacillariophyceae Haeckel, 1878

**Order Rhopalodiales D.G.Mann, 1990**

#### ﻿Family Rhopalodiaceae (Karsten) Topachevs’kyi & Oksiyuk, 1960

##### Genus *Epithemia* Kützing, 1844

**282. *Epithemiaadnata* (Kützing) Brébisson, 1838: 16.**

*Epithemiazebra* (Ehrenberg) Ehrenberg, 1838: 191, pl. 14 fig. 7.

Epithemiazebravar.genuina Grunow nom inval., 1862: 328.

**Observation.** Main basin: Gasse (1986), Cocquyt and De Wever (2002), Stoof-Leichsenring et al. (2011), Stoof-Leichsenring et al. (2012), NM91.1-S, NM93.1-S.

Crescent Island Crater: Richardson and Richardson (1972), Cocquyt and De Wever (2002), NC20.

Lake Oloidien: Gasse (1986).

Lake Sonachi: Bachmann (1938), NS93.2-F.

**Occurrence.** Plankton, bottom mud, sediment core.

Gasse (1986) reported also *Epithemiaporcellus* Kützing, 1844 (as Epithemiazebravar.porcellus (Kützing) Grunow, 1862) and Epithemiaadnatavar.saxonica (Kützing) R.M.Patrick, 1975 (as Epithemiazebravar.saxonica (Kützing) Grunow, 1862) from East Africa but it cannot be deduced from the publication if these two taxa were observed in the samples from Lake Naivasha and it satellite lakes.

**283. *Epithemiaargus* (Ehrenberg) Kützing, 1844: 35, pl. 22 figs 55, 56.**

**Observation.** Crescent Island Crater: Gasse (1986), core NC20.

**Occurrence.** Plankton, sediment core.

**284. Epithemiaargusvar.intermedia (Hilse) Ant.Mayer, 1936: 99, pl. 6 figs 13, 14, 16, pl. 7 fig. 5.**

*Epithemiaintermedia* Hilse, 1860: 76.

**Observation.** Crescent Island Crater: Richardson and Richardson (1972).

**Occurrence.** Sediment core.

**285. *Epithemiahyndmannii* W.Smith, 1850: 124.**

**Observation.** Lake Oloidien: Gasse (1986).

**Occurrence.** Plankton.

**286. *Epithemiaporcellus* Kützing, 1844: 34, pl. 5 figs 18, 19.**

Epithemiazebravar.porcellus (Kützing) Grunow, 1862: 328, pl. 3 figs 3, 4.

**Observation.** Main basin: Rich (1932).

**Occurrence.** Bottom mud.

**287. *Epithemiasorex* Kützing, 1844: 33, pl. 5/12 figs 5a–c.**

**Observation.** Main basin: Bachmann (1938), Stoof-Leichsenring et al. (2012), NM91.1-S.

Crescent Island Crater: Richardson and Richardson (1972), NC20.

Lake Oloidien: Gasse (1986).

**Occurrence.** Plankton, littoral, sediment core.

**288. *Epithemiaturgida* (Ehrenberg) Kützing, 1844: 34, pl. 5 fig. 14.**

**Observation.** Main basin: Rich (1932).

Crescent Island Crater: Richardson and Richardson (1972).

**Occurrence.** Bottom mud, sediment core.

**289. Epithemiaturgidavar.capitata Fricke, 1904: pl. 250 fig. 7.**

**Observation.** Main basin: Bachmann (1938).

**Occurrence.** Littoral.

**290. *Epithemia* sp.**

**Observation.** Crescent Island Crater: Richardson and Richardson (1972).

**Occurrence.** Sediment core.

##### Genus *Rhopalodia* O.Müller, 1895

**291. *Rhopalodiagibba* (Ehrenberg) O.Müller, 1895: 65, pl. 1 figs 15–17.**

**Observation.** Main basin: Gasse (1986), Stoof-Leichsenring et al. (2011), Stoof-Leichsenring et al. (2012), NM91.1-S, NM93.1-S.

Crescent Island Crater: Richardson and Richardson (1972), Gasse (1986), NC20.

Lake Oloidien: Gasse (1986).

**Occurrence.** Bottom mud, sediment core.

**292. Rhopalodiagibbavar.ventricosa (Kützing) H.Peragallo & M.Peragallo, 1900: 302, pl. 77 figs 3–5.**

*Epithemiaventricosa* Kützing, 1844: 35, pl. 30 fig. 9a, b.

*Rhopalodiaventricosa* (Kützing) O.Müller, 1895: 65, pl. 1 figs 20, 21.

**Observation.** Main basin: Rich (1932).

Lake Sonachi: Rich (1932).

**Occurrence.** Plankton, bottom mud.

**293. *Rhopalodiagibberula* Mereschkowsky, 1906: 32, fig. 19.**

**Observation.** Main basin: Gasse (1986), Cocquyt and De Wever (2002), NM91.1-S, NM93.1-S.

Crescent Island Crater: Richardson and Richardson (1972), Cocquyt and De Wever (2002), NC20.

Lake Oloidien: Gasse (1986).

Lake Sonachi: Gasse (1986), Cocquyt and De Wever (2002), NS93.2-F.

**Occurrence.** Plankton, bottom mud, sediment core.

**294. Rhopalodiagibberulavar.vanheurckii O.Müller, 1900: 32, pl. X fig. 11, pl. XI figs 6, 7.**

**Observation.** Crescent Island Crater: NC20.

**Occurrence.** Sediment core.

**295. *Rhopalodiaparallela* (Grunow) O.Müller, 1895: 64, pl. 1 figs 13, 14.**

Epithemiagibbavar.parallela Grunow, 1862: 327, pl. 6 fig. 7.

*Epithemiaparallela* (Grunow) Ruck & Nakov, 2016b: 1.

**Observation.** Main basin: Rich (1932).

**Occurrence.** Bottom mud.

**296. *Rhopalodiarhopala* (Ehrenberg) Hustedt, 1949: 128.**

*Epithemiarhopala* (Ehrenberg) Cocquyt & R.Jahn, 2018: 51, figs 51–58.

**Observation.** Crescent Island Crater: NC20.

**Occurrence.** Sediment core.

**297. *Rhopalodiavermicularis* O.Müller, 1895: 67, pl. I figs 34–39, pl. II figs 10, 11, 14.**

*Epithemiavermicularis* (O.Müller) Cocquyt & R.Jahn, 2018: 50, figs 47–50.

**Observation.** Crescent Island Crater: Richardson and Richardson (1972).

Lake Oloidien: Gasse (1986).

**Occurrence.** Bottom mud, sediment core.

### ﻿Class Bacillariophyceae Haeckel, 1978

**Order Surirellales D.G.Mann, 1990**

#### ﻿Family Surirellaceae Kützing, 1844

##### Genus *Cymatopleura* W.Smith, 1851

**298. Cymatopleuracf.clavata (O.Müller) Cocquyt & R.Jahn, 2014: 413.**

Cymatopleurasoleavar.clavata O.Müller, 1904: 22, fig. 1.

*Surirellaclavata* (O.Müller) Cocquyt & R.Jahn, 2017: 98.

**Observation.** Main basin: Stoof-Leichsenring et al. (2011), Stoof-Leichsenring et al. (2012).

Crescent Island Crater: NC20.

**Occurrence.** Sediment core.

**299. Cymatopleuracf.elliptica (Brébisson) W.Smith, 1851: 13, pl. 3 figs 10, 11.**

*Surirellaelliptica* Brébisson ex Kützing, 1844: 61, pl. 28 fig. 28.

**Observation.** Crescent Island Crater: Richardson and Richardson (1972).

**Occurrence.** Sediment core.

**300. *Cymatopleuralibrile* (Ehrenberg) Ehrenberg, 1845: 139.**

*Cymatopleurasolea* (Brébisson) W.Smith, 1851: 12, pl. 3 fig. 9.

*Surirellasolea* (Brébisson) Brébisson, 1838: 17.

**Observation.** Main basin: Owino et al. (2020).

Crescent Island Crater: Richardson and Richardson (1972).

**Occurrence.** Sediment core.

**Remark.**Rich (1932) reported a *Cymatopleura* sp. from bottom mud in the main basin of Lake Naivasha, probably one of the taxa mentioned above.

##### Genus *Iconella* Jurilj, 1949

**301. *Iconellabiseriata* (Brébisson) Ruck & Nakov, 2016: 1.**

*Surirellabiseriata* Brébisson, 1835: 53, pl. VII.

**Observation.** Main basin: Stoof-Leichsenring et al. (2011), Stoof-Leichsenring et al. (2012).

**Occurrence.** Sediment core.

**302. *Iconellaengleri* (O.Müller) Cocquyt & R.Jahn, 2017: 87.**

*Surirellaengleri* O.Müller, 1903: 28, pl. I fig. 4.

Surirellaenglerif.angustior O.Müller, 1903: 28, pl. I fig. 5.

**Observation.** Main basin: Bachmann (1938), Gasse (1986).

**Occurrence.** Plankton.

**303. *Iconellafuellebornii* (O.Müller) Cocquyt & R.Jahn, 2017: 88.**

*Surirellafuellebornii* O.Müller, 1904: 30.

**Observation.** Crescent Island Crater: Richardson and Richardson (1972).

**Occurrence.** Sediment core.

**304. *Iconellalinearis* (W.Smith) Ruck & Nakov, 2016: 2.**

*Surirellalinearis* W.Smith, 1853: 31, pl. VIII fig. 58.

**Observation.** Main basin: Rich (1932), Owino et al. (2020), NM91.1-S, NM93.1-S.

Crescent Island Crater: NC20.

**Occurrence.** Bottom mud, sediment core.

**305. Iconellalinearisvar.elliptica (O.Müller) Cocquyt & R.Jahn, 2017: 90.**

Surirellalinearisvar.elliptica O.Müller, 1904: 30, pl. 1 fig. 10.

**Observation.** Main basin: Rich (1932).

**Occurrence.** Bottom mud.

**306. *Iconellanervosa* (A.W.F.Schmidt) Cocquyt & R.Jahn, 2017: 93.**

Surirellateneravar.nervosa A.W.F.Schmidt, 1875: pl. 23 figs 15–17.

*Surirellanervosa* (A.W.F.Schmidt) Ant.Mayer 1913: 341, pl. 23 fig. 5, pl. 28 figs 8, 9.

**Observation.** Crescent Island Crater: NC20.

**Occurrence.** Sediment core.

**307. *Iconellanyassae* (O.Müller) Cocquyt & R.Jahn, 2017: 93.**

*Surirellanyassae* O.Müller, 1904: 33, pl. II fig. 3.

**Observation.** Crescent Island Crater: Richardson and Richardson (1972).

**Occurrence.** Sediment core.

**308. Iconellacf.tenera (W.Gregory) Ruck & Nakov, 2016: 2.**

*Surirellatenera* W.Gregory, 1856: 11, pl. 1 fig. 38.

**Observation.** Main basin: NM93.1-S.

Sediment core.

**309. *Iconella* sp.**

**Observation.** Main basin: NM91.1-S, NM93.1-S.

**Occurrence.** Sediment core.

##### Genus *Surirella* Turpin, 1828

**310. *Surirellaovalis* Brébisson**, 1838: 17.

**Observation.** Main basin: Owino et al. (2020).

Crescent Island Crater: NC20.

**Occurrence.** Sediment core.

**Remark.**Owino et al. (2020) also reported a *Surirella* sp. but we do not know if it concerns a *Surirellasensu stricto* or a *Iconella* species.

## ﻿Conclusions

The diatom flora of Lake Naivasha and its satellite lakes is highly diverse with 310 species and infraspecific taxa reported to date. This number will certainly increase as many taxa remain unidentified, and some species and infraspecific taxa were lumped when older taxonomy was used for identification. Moreover, it is well known that the more material is studied, the more species and infraspecific taxa will be observed, especially rare taxa. In addition, the present species richness is based only on morphological characteristics. Only a few studies exist at present on the molecular identity of the diatoms observed in tropical Africa. Available genetic data appears to be inadequate for most of the tropical African diatoms as shown by the results of Stoof-Leichsenring et al. (2012) where only less than 30% (14 out of the 49 species) could be more or less linked to a species present in the existing molecular library.

Extensive molecular investigation of the diatoms in Lake Naivasha and it satellite lakes, and in tropical Africa in general, would provide more information on the identity of the observed species and their endemic or cosmopolitan nature. We are convinced that the proportion of reported diatom species with a distribution restricted to tropical Africa (3.4%), to the African continent (1.0%), or to the pantropics (2.0%) based on the present checklist, will increase when molecular data of the observed taxa become available.

The checklist presented here will certainly provide a useful baseline for further diatom research in Kenya, and more generally in tropical Africa, in order to align molecular and morphological identifications.
